# Associations between total and regional fat-to-muscle mass ratio and fracture risk in elderly population: a prospective cohort study in UK Biobank

**DOI:** 10.3389/fmed.2026.1830114

**Published:** 2026-06-24

**Authors:** Kaijie Guo, Ruiying Zhang, Xiurui Zhang, Kexin Zhang, Guangtao Fu, Yuanchen Ma

**Affiliations:** 1 Department of Orthopedics, Guangdong Provincial People's Hospital (Guangdong Academy of Medical Sciences), Southern Medical University, Guangzhou, China; 2 Guangdong Cardiovascular Institute, Guangdong Provincial People's Hospital, Guangzhou, China

**Keywords:** body composition, elderly fracture, fat-to-muscle mass ratio, prospective cohort study, UK Biobank

## Abstract

**Background:**

Previous studies reported that the overall risk of fragility fractures increased with both obesity (high body mass index, BMI) and sarcopenia (low BMI) in elderly population, which highlights that BMI may not fully capture the crucial role of skeletal muscle and fat tissue distribution. The total and regional fat-to-muscle mass ratio (FMR), which consider the interaction of both muscle mass and fat tissue, might better reflect the body composition variations associated with fragility fractures.

**Methods:**

We included 211,881 participants aged ≥60 years from the UK Biobank who had complete bioelectrical impedance analysis-derived body composition data. FMR was calculated as fat mass divided by muscle mass. Incident osteoporotic fractures (OF) and major osteoporotic fractures (MOF) were recorded and multivariable Cox models assessed associations between total/regional FMR and fracture risk, followed by sex-stratified analyses. Non-linear relationships were examined using restricted cubic splines.

**Results:**

Among 12,274 participants with incident MOF, arms and trunk FMR showed the strongest positive associations with MOF risk after adjustment for BMI, bone mineral density, sex, and other confounders. Compared to the lowest quintile (First quintile, Q1), the highest quintile (Fifth quintile, Q5) of arms FMR was associated with a 24% increased risk of MOF, and trunk FMR with a 16% increase. Non-linear associations between arms and trunk FMR and fractures were observed (*P* < 0.05). In contrast, legs FMR above 0.453 exhibited a protective effect with reaching a significant level (HR = 0.92). When considering the subgroup analysis, a significant sex interaction was found (P-interaction < 0.05): the MOF risk per Q5 vs. Q1 was higher in men (HR = 1.50, 95% CI: 1.26–1.77 for arms; HR = 1.26, 1.07–1.47 for trunk) than in women (HR = 1.22, 1.05–1.41 for arms; HR = 1.15, 1.03–1.27 for trunk). Legs FMR was inversely associated with MOF in men and women (Q4 vs. Q1: HR = 0.85, 0.74–0.98 in men; HR = 0.87, 0.78–0.97 in women). Results for OF showed similar trends.

**Conclusions:**

Our findings indicate that elevated FMR in the arms and trunk is associated with an increased risk of MOF and OF, independent of sex, BMI, and BMD. While further studies are needed to formally compare its predictive performance with traditional metrics like BMI, region-specific FMRs may serve as a potential complementary tool for assessing fracture risk in the elderly.

## Introduction

The rising burden of fractures among the aging population represents a growing public health challenge due to the accelerated urbanization and extended life expectancy (1). Approximately 60% of women and nearly a quarter of men aged 50 and above are expected to sustain fragility fractures in Korea (2). The worldwide annual incidence of osteoporosis-related fractures is estimated to reach 3 million in 2025 (3). The one-year mortality rate following hip fracture varied from 16.6% to 23.9% according to different study designs (4, 5). Moreover, a substantial proportion of patients fail to regain their pre-fracture level of independence (6). Consequently, it is imperative to proactively identify patients at high risk of fractures, who require essential medical interventions for secondary fracture prevention.

Body mass index (BMI) is a well-documented risk factor for fracture in the elderly population (7), which is also incorporated as an input variable for risk prediction in FRAX (Fracture Risk Assessment Tool), the most widely used fracture risk assessment tool worldwide (8). However, a recently published meta-analysis including a total of 1,667,922 men and women from 32 countries (63 cohorts) demonstrated that both low and high BMI are associated with a greater risk of fracture regardless of adjustment for BMD (Bone mineral density) (9). The U-shaped relationship between BMI and fragility fracture risk suggests the limited predictive value of BMI alone and the necessity for further investigation of body composition analysis. Additionally, several studies (10–12) have documented the complex relationships between fat mass and lean mass in the determination of fracture risk regardless of falls, implicating the involvement of muscle- and adipose tissue- related metabolic dysregulation beyond mechanical factors.

The fat-to-muscle mass ratio (FMR) is calculated by dividing total or regional fat mass by total or regional muscle mass, which is typically derived from bioelectrical impedance analysis (BIA) or dual-energy X-ray absorptiometry (DXA) (13). Considering the interaction and distribution of both muscle mass and fat tissue, FMR has been shown to be closely associated with musculoskeletal disorders including knee osteoarthritis (14). As both skeletal muscle and fat tissue are closely related to fragility fracture, the total and regional FMR may better reflect the body composition profiles associated with fracture risk compared to BMI alone.

In this prospective cohort study using UK Biobank data, we aimed to investigate the associations of total and regional FMR with the incidence of fragility fractures in older adults, utilizing multivariable Cox proportional hazards models and restricted cubic splines for non-linearity analysis.

## Methods and materials

### Study population

**Figure 1:**
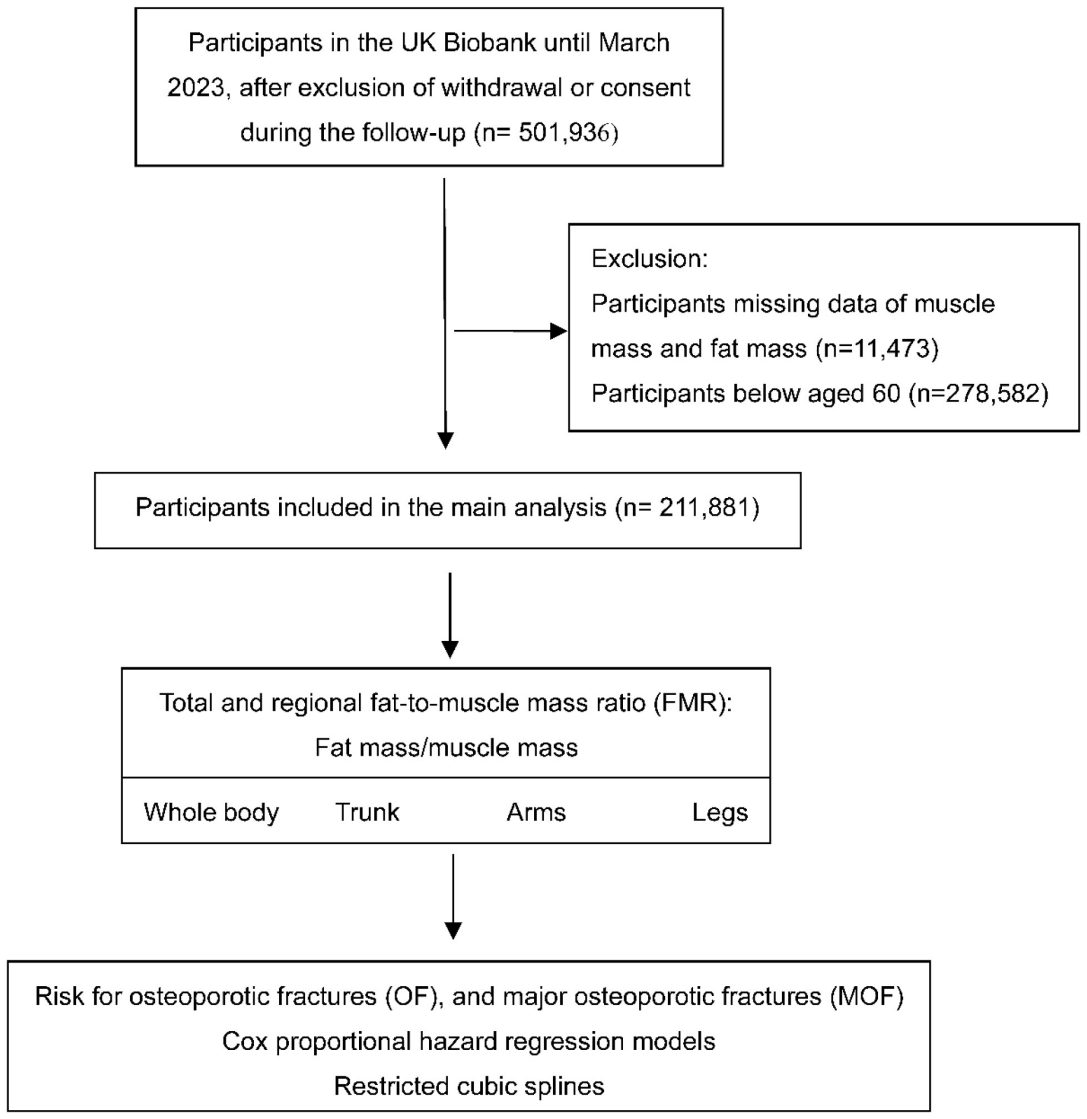
Flowchart of study participant selection from the UK Biobank. The flowchart details the exclusion process of participants from the UK Biobank. After excluding individuals with missing body composition data (*n* = 11,473) and those under 60 years of age (*n* = 278,582), a total of 211,881 participants remained for the final analysis. FMR was calculated for the whole body and regional areas (trunk, arms, and legs). Associations with OF and MOF risk were assessed using Cox models and restricted cubic splines. **FMR**: fat-to-muscle mass ratio; **MOF**: major osteoporotic fracture; **OF**: osteoporotic fracture.

UK Biobank is a robust, ongoing prospective cohort study that enrolled over 500,000 participants aged 40–69 years across 22 assessment centers in the United Kingdom between 2006 and 2010. At baseline, participants underwent standardized physical and functional assessments, and biological samples were collected for comprehensive genotyping and biochemical profiling. Ethical approval was granted by the North West Multi-centre Research Ethics Committee (Ref: 11/NW/0382). This study was performed under UK Biobank application No. 849333, with outcome data obtained through integration with National Health Service (NHS) electronic health records. After excluding participants with missing data on fat mass or muscle mass (*n* = 11,473) and those aged under 60 years at baseline (*n* = 278,582), a final sample of 211,881 participants was included in the primary analysis (Figure 1).

### Exposure assessments

Following a standardized protocol, height was measured using a Seca device, while weight, BMI and segmental body composition were estimated via an eight-contact electrode Tanita BC418MA bioelectrical impedance analyzer (Tanita, Tokyo, Japan). This validated BIA approach provided precise data on fat and estimated muscle mass across the whole body and specific regions (trunk, arms, and legs). Notably, participants with physical contraindications, such as pacemakers or amputations, were excluded to ensure data reliability (more details can be found at https://biobank.ndph.ox.ac.uk/ukb/label.cgi?id=100009). Consistent with established methodologies in body composition research, FMR was calculated by dividing the fat mass by the predicted muscle mass of the corresponding part and categorized into quintiles (Q1–Q5).

### Outcome assessment

The primary outcomes were the first incident osteoporotic fracture (OF) and major osteoporotic fracture (MOF) following study inclusion. OF was defined as any fracture consistent with an osteoporotic origin (clinical spine, humerus, wrist, pelvis, ribs, and femur), excluding high-impact trauma sites (craniofacial, cervical spine, hands, and feet). MOF was specifically defined as a subset of fractures occurring at four key sites: the clinical spine, proximal femur, proximal humerus, and wrist. Events were identified through rigorous integration of ICD-10 codes from hospital inpatient records (Hospital Episode Statistics for England, Scottish Morbidity Record for Scotland, and Patient Episode Database for Wales) and self-reported data (Supplementary Table S1). To ensure accuracy and avoid duplication, event dates were defined as the earliest occurrence across all available sources. In cases of temporal overlap between sources, priority was assigned to ICD-10 hospital records for their diagnostic precision. Participants were followed from enrollment (2006–2010) until the first occurrence of a fracture, death, loss to follow-up, or the administrative censoring date (May 31, 2024, for England and Wales; December 31, 2023, for Scotland).

### Covariates

Based on prior knowledge and FRAX criteria, we adjusted for potential confounders. Sociodemographic factors included age, sex, ethnicity (White or others), education level (higher or lower), and Townsend Deprivation Index quintiles. Health behaviors included smoking status, alcohol consumption frequency (never/occasional, 1–2, 3–4 times/week, or daily), physical activity, serum vitamin D, and dietary habits (beef and processed meat intake). Clinical and anthropometric characteristics included BMI (kg/m^2^, continuous and categorical), estimated bone mineral density (eBMD) T-scores, history of glucocorticoid use, rheumatoid arthritis, and diabetes mellitus. For women, reproductive factors (history of bilateral oophorectomy and use of hormone replacement therapy, HRT) were considered. Finally, fracture-related risks, including falls in the last year (0, 1, or ≥2) and history of fracture within the previous 5 years, were incorporated.

### Statistical analyses

Baseline characteristics of participants, stratified by incident OF events, were presented as mean (standard deviation, SD) for continuous variables and as frequencies (percentages) for categorical variables. Inter-group differences were compared using Student's *t*-tests or chi-square tests, as appropriate.

Cox proportional hazards models were used to estimate the hazard ratios (HRs) and 95% confidence intervals (CIs) for the associations between quintiles of FMR in the whole body and specific regions (trunk, arms, and legs) and incident OF and MOF. The initial model (Model 1) was adjusted for age, gender, race, Townsend deprivation index, education, smoking status, alcohol intake frequency, beef intake frequency, processed meat intake, serum vitamin D, glucocorticoids use at baseline, falls in the last year, fractured or broken bones in last 5 years, diabetes history and rheumatoid arthritis history. Model 2 was adjusted for BMI categories. To further examine whether the FMR-fracture association persisted independent of overall adiposity and bone mineralization status, Model 3 was further adjusted for both BMI and eBMD. The schoenfeld residuals method was used to test proportional hazards assumption, and no significant violations were observed.

We used the restricted cubic spline (RCS) curves with four knots placed at the 20th, 40th, 60th, and 80th percentiles to investigate potential non-linear relationships between FMR and incident fractures. If the relationship was non-linear, we used a recursive algorithm to calculate the inflection points between FMR and fractures. A two-segment Cox proportional hazards model was used on both sides of the inflection point to investigate the association between FMR and incident fractures. Given that body composition components, such as fat and muscle mass, are markedly different between men and women, subgroup analyses were performed and interactions between FMR and sex were evaluated using multiplicative interaction terms.

Several sensitivity analyses were performed to ensure the robustness of our findings, including: (1) utilizing multiple imputation to address missing covariate data; (2) excluding participants who experienced fracture events within the first 2 years of follow-up to minimize potential reverse causality; (3) additionally adjusting for history of HRT use among female participants; (4) further adjusting for physical activity; (5) restricting the outcome definitions to hospital-record-only fractures; and (6) applying Fine-Gray competing-risk models. A two-sided *P* < 0.05 was considered statistically significant. All analyses were performed using R software (version 4.4.0).

## Results

### Baseline characteristics

A total of 211,881 UK Biobank participants were included. Over a median 15.0 years of follow-up, we recorded 18,593 incident OF and 12,274 MOF. Baseline characteristics of the participants, stratified by the occurrence of OF, are presented in Table 1. Participants who developed OF were more likely to be older and female, of White ethnicity and have higher levels of socioeconomic deprivation. Compared to those without OF, they tended to have lower education levels, a higher proportion of current smokers, and a lower frequency of regular alcohol intake. Individuals with incident OF had significantly lower BMI, serum vitamin D levels, and eBMD T-scores. Furthermore, they were more likely to have a history of glucocorticoids use, rheumatoid arthritis, diabetes, and a higher frequency of falls or previous fractures within the last 5 years.

**Table 1:** Baseline characteristics in the global population.

Characteristic	No-OF	OF	*P*-value
	(*n* = 193,288)	(*n* = 18,593)	
Age (years)	64.09 ± 2.84	64.57 ± 2.90	< 0.01^*^
Female (%)	99,475 (51.5)	12,677 (68.2)	< 0.01^*^
White (%)	186,524 (96.9)	18,189 (98.3)	< 0.01^*^
**Townsend Deprivation Index (%)**			
1st	41,996 (21.7)	3,664 (19.7)	< 0.01^*^
2	41,932 (21.7)	3,783 (20.4)	
3	40,197 (20.8)	3,774 (20.3)	
4	36,366 (18.8)	3,673 (19.8)	
5th	32,630 (16.9)	3,688 (19.8)	
Higher education (%)	108,282 (57.2)	9,849 (54.4)	< 0.01^*^
**Smoking status (%)**			
Never	96,168 (50.1)	9,232 (50.0)	< 0.01^*^
Former	80,459 (41.9)	7,471 (40.4)	
Current	15,508 (8.1)	1,779 (9.6)	
Physical activities (MET-min/week)	2,783.52 ± 2,673.76	2,744.13 ± 2,647.61	0.104
**Alcohol intake frequency (%)**			
Never or occasional	58,449 (30.3)	6,302 (34.0)	< 0.01^*^
1–2 times a week	46,331 (24.0)	4,240 (22.9)	
3–4 times a week	42,983 (22.3)	3,742 (20.2)	
Daily or almost daily	45,195 (23.4)	4,262 (23.0)	
Serum Vitamin D (nmol/L)	49.90 [35.50, 64.50]	49.00 [34.10, 64.40]	< 0.01^*^
BMI (kg/m^2^)	27.64 ± 4.51	27.25 ± 4.72	< 0.01^*^
**BMI Categories (%)**			
Underweight	636 (0.3)	119 (0.6)	< 0.01^*^
Normal weight	54,763 (28.3)	6,295 (33.9)	
Overweight	88,694 (45.9)	7,683 (41.3)	
Obesity	49,195 (25.5)	4,496 (24.2)	
eBMD T-scores	−0.40 ± 1.24	−0.93 ± 1.13	< 0.01^*^
Glucocorticoids use (%)	2,680 (1.4)	418 (2.2)	< 0.01^*^
**Beef Intake Frequency (%)**			
Never or less than once a week	105,586 (54.9)	10,306 (55.8)	< 0.01^*^
Once a week	63,408 (33.0)	5,811 (31.4)	
2 times a week or more	23,309 (12.1)	2,361 (12.8)	
**Processed Meat Intake (%)**			
Never or less than once a week	76,417 (39.7)	7,964 (43.0)	< 0.01^*^
Once a week	57,777 (30.0)	5,460 (29.5)	
2 times a week or more	58,532 (30.4)	5,099 (27.5)	
**Falls in the last year (%)**			
0	152,061 (78.9)	12,928 (69.8)	< 0.01^*^
1	28,132 (14.6)	3,514 (19.0)	
≥2	12,486 (6.5)	2,082 (11.2)	
Fractured or broken bones in last 5 years (%)	17,376 (9.0)	3,205 (17.3)	< 0.01^*^
Fractures resulting from simple falls (%)	11,806 (68.9)	2,327 (73.6)	< 0.01^*^
Rheumatoid arthritis history (%)	3,011 (1.6)	481 (2.6)	< 0.01^*^
Diabetes history (%)	17,958 (9.3)	1,931 (10.4)	< 0.01^*^

Data are presented as mean ± SD, median [IQR], or *n* (%).

**OF**, osteoporotic fractures; **eBMD**, estimated bone mineral density; **BMI**, body mass index; **SD**, standard deviation; **IQR**, interquartile range; **MET**, metabolic equivalent of task.

^*^P < 0.05 was considered statistically significant.

### FMRs and incident fractures outcomes

**Figure 2:**
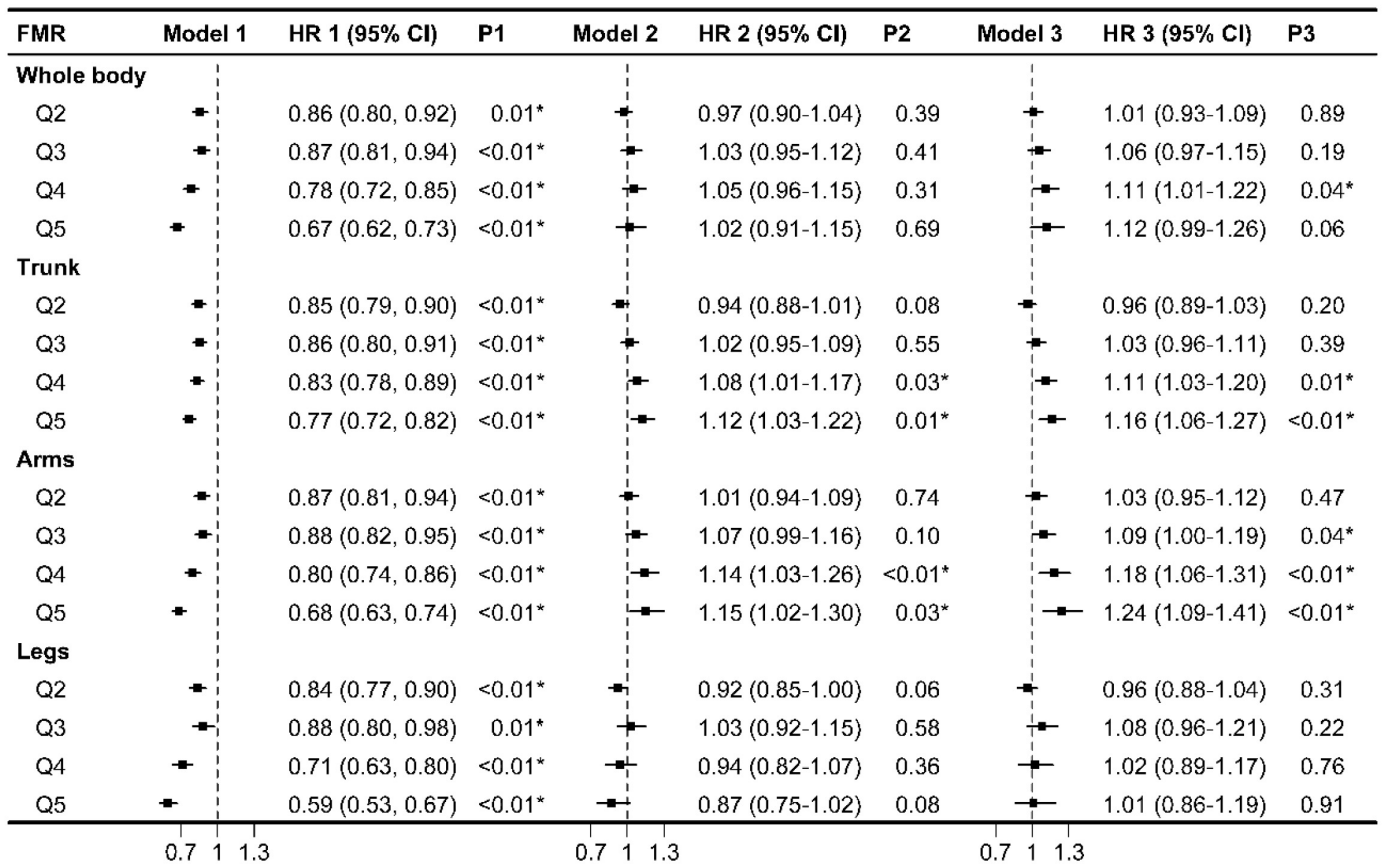
Cox proportional hazard regression analyses on associations between FMR indices and risk of incident MOF. The forest plots illustrate the HRs and 95% CIs for the associations between FMR indices (Whole body, Trunk, Arms, and Legs) and the risk of incident MOF. Model 1 was adjusted for age, gender, race, Townsend deprivation index, education, smoking status, alcohol intake frequency, beef intake, processed meat intake, serum vitamin D, glucocorticoid use, falls in the last year, prior fractures, diabetes, and rheumatoid arthritis. Model 2 was further adjusted for BMI categories. Model 3 was further adjusted for both BMI categories and eBMD T-scores. “*” indicates statistical significance with *P* < 0.05. **FMR**, fat-to-muscle mass ratio; **MOF**, major osteoporotic fractures; **eBMD**, estimated bone mineral density; **BMI**, body mass index; **HR**, hazard ratio; **CI**, confidence interval.

Figure 2 and Supplementary Figure S1 illustrate the associations between site-specific FMRs and fracture risk. Notably, the initially observed protective effect of higher FMR was reversed into a risk-increasing effect following adjustment for BMI. In the final model, adjusting for BMD clearly strengthened these associations, which remained consistently significant across both OF and MOF analyses.

Among the various body parts, arms and trunk FMRs exhibited the strongest positive associations with MOF risk. Specifically, compared with the lowest quintile (Q1), the highest quintile (Q5) of FMR in the arms was associated with a 24% higher risk of MOF (adjusted HR, 1.24; 95% CI, 1.09–1.41), while Q5 of trunk FMR was associated with a 16% higher risk (adjusted HR, 1.16; 95% CI, 1.06–1.27). Similarly, significant associations were observed for OF, with corresponding HRs of 1.20 (95% CI, 1.08–1.33) for the arms and 1.11 (95% CI, 1.03–1.20) for the trunk. Regarding whole-body FMR, although the results were not statistically significant, a clear rising trend in adjusted HRs was observed from Q1 through Q5.

### Non-linear associations between FMRs and fractures

**Figure 3:**
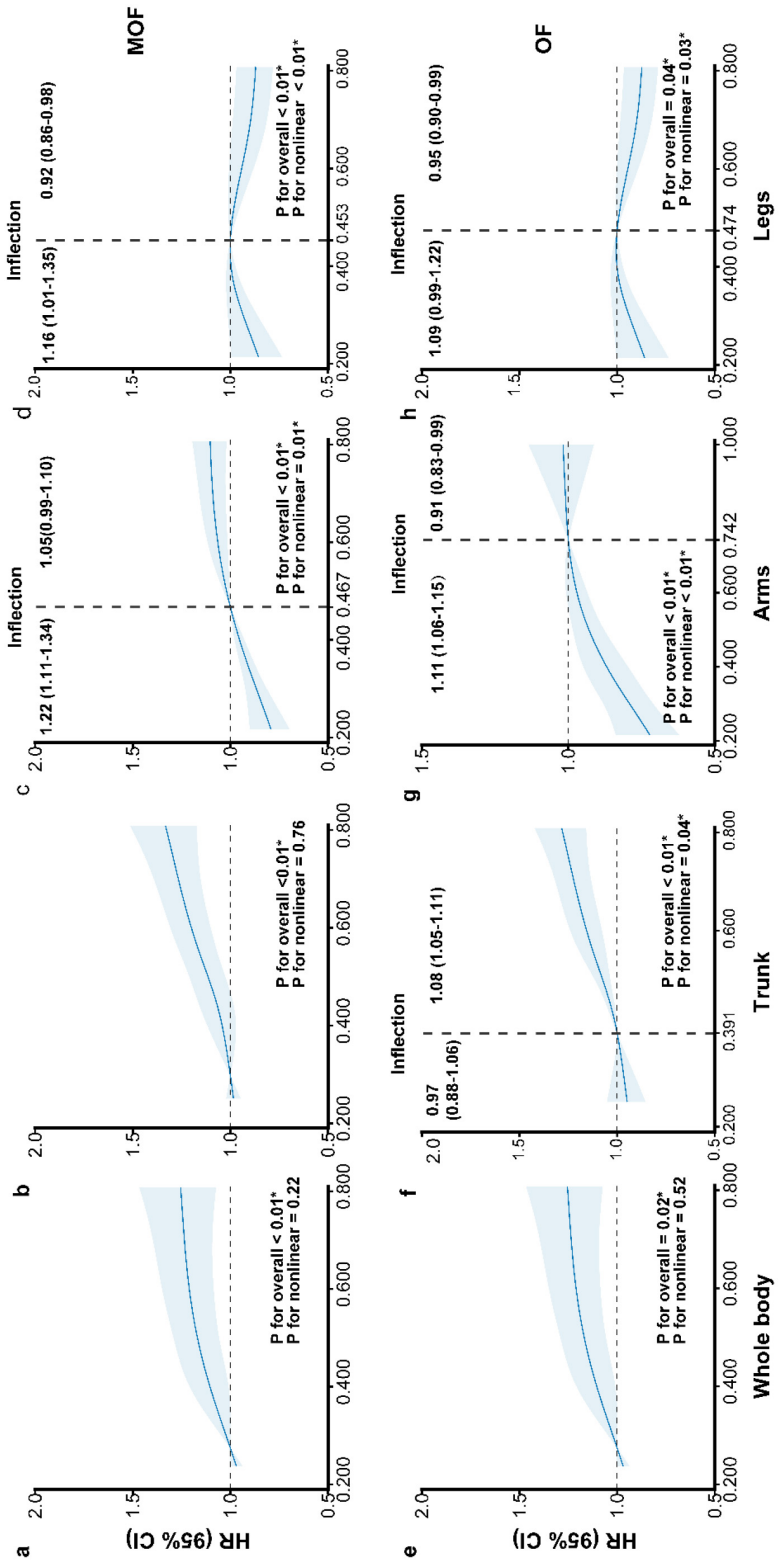
Restricted cubic spline curves for associations of FMR with MOF **(a–d)** and OF **(e–h)**. Restricted cubic spline curves illustrate the associations of FMR with the risk of MOF **(a–d)** and OF **(e–h)**. Solid blue lines and shaded areas represent HRs and 95% CIs. Vertical dashed lines indicate inflection points. The model adjusted for age, race, socioeconomic status, lifestyle factors, clinical history (diabetes, rheumatoid arthritis), medication use, falls, BMI categories, and eBMD T-scores. P-overall and *P*-non-linearity are shown for each panel. **FMR**, fat-to-muscle mass ratio; **MOF**, major osteoporotic fractures; **OF**, osteoporotic fractures; **eBMD**, estimated bone mineral density; **BMI**, body mass index; **HR**, hazard ratio; **CI**, confidence interval.

In the restricted cubic spline analysis (Figure 3), we visualized the non-linear associations between site-specific FMRs and incident fractures. Among the regional body parts, arms FMR exhibited a rapid increase in the risk of both MOF and OF until reaching the inflection points (0.467 for MOF and 0.742 for OF), after which the trend leveled off (*P*-non-linearity < 0.05). Below these inflection points, the HRs per 1-SD increase in arms FMR were 1.22 (95% CI: 1.11–1.34) for MOF and 1.11 (95% CI: 1.06–1.15) for OF.

The risk of OF remained stable until a trunk FMR of 0.391, after which it rose sharply (*P* for non-linearity = 0.04). The HRs per 1-SD increase were 0.97 (95% CI, 0.88–1.06) below this threshold and increased to 1.08 (95% CI, 1.05–1.11) above it.

Notably, the associations of legs FMR with both MOF and OF were opposite to those observed in other regions. The risk remained relatively flat until reaching the inflection points (0.453 for MOF and 0.474 for OF), after which the fracture risk dropped rapidly, with HRs per 1-SD increase of 0.92 (95% CI: 0.86–0.98) for MOF and 0.95 (95% CI: 0.90–0.99) for OF.

### Subgroup analyses and sensitivity analyses

**Figure 4:**
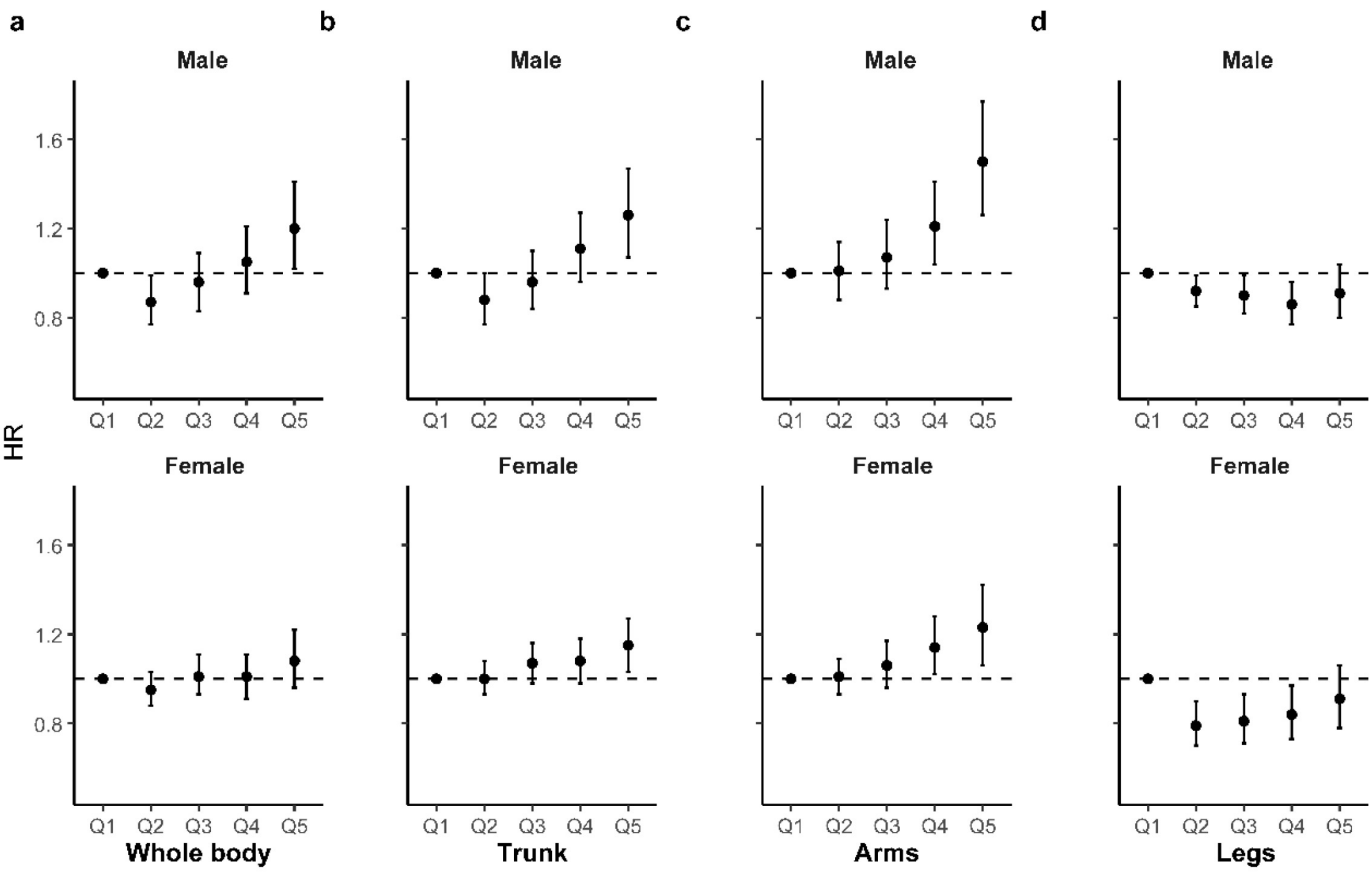
Forest plots of associations between FMR and MOF, stratified by sex. The forest plots show the HRs and 95% CIs for the associations between FMR indices and the risk of MOF, stratified by Male and Female. Participants were categorized into quintiles (Q1–Q5), with Q1 as the reference group. The model was adjusted for age, race, Townsend deprivation index, education, smoking status, alcohol intake frequency, beef and processed meat intake, serum vitamin D, glucocorticoid use, falls in the last year, prior fractures, diabetes, rheumatoid arthritis, BMI categories, and eBMD T-scores. **FMR**, fat-to-muscle mass ratio; **MOF**, major osteoporotic fractures **eBMD**, estimated bone mineral density; **BMI**, body mass index; **HR**, hazard ratio; **CI**, confidence interval.

Subgroup analyses by sex between FMR and MOF are presented in Figure 4 and Table 2. While overall trends remained consistent between men and women, significant interactions were observed (P for interaction < 0.05). Specifically, the positive associations of arms and trunk FMRs with MOF risk were more pronounced in men than in women (Q5 HR: 1.50 and 1.26 in men vs. 1.22 and 1.15 in women, respectively). For whole-body FMR, a significant risk-increasing effect was observed starting at Q4 for women (HR: 1.11, 95% CI: 1.01–1.22) and Q5 for men (HR: 1.20, 95% CI: 1.02–1.41).

**Table 2:** Cox proportional hazard regression analyses on associations between FMR indices and risk of incident MOF, stratified by sex.

FMR	HR	Model 3 (95% CI)	*P*	HR	Model 3 (95% CI)	*P*	*P* for interaction
		Women			Men		
Whole body							0.01^**^
Q2	1.01	(0.93, 1.09)	0.89	0.87	(0.77, 0.99)	0.03	
Q3	1.06	(0.97, 1.15)	0.19	0.96	(0.83, 1.09)	0.51	
Q4	1.11	(1.01, 1.22)	0.04^*^	1.05	(0.91, 1.21)	0.51	
Q5	1.12	(0.99, 1.26)	0.06	1.20	(1.02, 1.41)	0.03^*^	
Trunk							0.02^**^
Q2	1	(0.93, 1.08)	0.98	0.88	(0.77, 1.00)	0.04	
Q3	1.07	(0.98, 1.16)	0.13	0.96	(0.84, 1.10)	0.56	
Q4	1.08	(0.98, 1.18)	0.12	1.11	(0.96, 1.27)	0.16	
Q5	1.15	(1.03, 1.27)	< 0.01^*^	1.26	(1.07, 1.47)	< 0.01^*^	
Arms							0.01^**^
Q2	1.01	(0.93, 1.09)	0.83	1.01	(0.88, 1.14)	0.94	
Q3	1.06	(0.96, 1.17)	0.22	1.07	(0.93, 1.24)	0.33	
Q4	1.14	(1.02, 1.28)	0.02	1.21	(1.04, 1.41)	< 0.01^*^	
Q5	1.22	(1.05, 1.41)	< 0.01^*^	1.50	(1.26, 1.77)	< 0.01^*^	
Legs							0.09
Q2	0.92	(0.85, 0.99)	0.03	0.79	(0.70, 0.90)	< 0.01^*^	
Q3	0.90	(0.82, 0.99)	0.03	0.81	(0.71, 0.93)	< 0.01^*^	
Q4	0.87	(0.78, 0.97)	< 0.01^*^	0.85	(0.74, 0.98)	0.03^*^	
Q5	0.91	(0.80, 1.04)	0.15	0.91	(0.78, 1.06)	0.23	

Model 3 was adjusted for age, gender, race, Townsend deprivation index, education, smoking status, alcohol intake frequency, beef intake, processed meat intake, serum vitamin D, glucocorticoid use, falls in the last year, prior fractures, diabetes, and rheumatoid arthritis, BMI categories and eBMD T-scores.

**FMR**, fat-to-muscle mass ratio; **MOF**, major osteoporotic fractures; **eBMD**, estimated bone mineral density; **BMI**, body mass index; **HR**, hazard ratio; **CI**, confidence interval.

^*^P < 0.05 was considered statistically significant.

^**^P for interaction < 0.05 was considered statistically significant in the multiplicative interaction.

Conversely, significant inverse associations were observed between legs FMR and MOF risk in men and women. Compared with Q1, the Q4 of legs FMR was associated with a 15% reduction in MOF risk for men (HR: 0.85; 95% CI: 0.74–0.98) and a 13% reduction for women (HR: 0.87; 95% CI: 0.78–0.97). Similar but slightly weaker associations were observed for OF in men and women (Supplementary Figure S2 and Supplementary Table S4).

In sensitivity analyses (Supplementary Figure S3–7, Supplementary Table S5), consistent results were obtained when we: (1) utilized imputed data; (2) excluded participants with fracture events during the first 2 years of follow-up to minimize reverse causality; (3) additionally adjusted for history of bilateral oophorectomy and HRT use in women; (4) further adjusted for physical activity; (5) restricted the outcomes to hospital-record-only fractures; or (6) applied Fine-Gray competing-risk models. These robust results consistently supported the primary associations observed.

## Discussion

Previous studies have well proven that both low and high BMI are related to increased risk of fragility fractures (7, 9, 15). Interestingly, they also reported significant differences of fracture sites and sex between patients in the low and high BMI groups, which highlights the potential further mechanistic implications, especially the distribution and location of adipose and muscle tissue. To our knowledge, this is the first large-scale prospective study to examine the longitudinal associations of both total and regional FMR with the risk of fragility fractures. We also believed that our results provided adequate strength of evidence due to the large volume of sample size (over 211,881 participants derived from UK Biobank).

In the present study, we found that elevated arms and trunk FMR were significantly associated with increased risk of MOF and OF regardless of sex and BMD in elderly population, suggesting that greater trunk adiposity and decreased muscle mass may predispose individuals to fragility fractures. Consistent with our results, a Meta-Analysis with a total sample size of 295,674 individuals reported that abdominal obesity (defined by various waist-hip ratios) was positively associated with the risk of hip fracture in men and women, even after adjustment for BMI (16). Previous studies well documented that inflammatory cytokines (including CRP, TNF-α, IL-1, and IL-6) that released by visceral adipose tissue cause bone remodeling imbalance, with enhanced bone reabsorption and inhibited bone formation (17–19). It was also reported that abdominal obesity causes instability and impaired balance, subsequently increases the risk of falling and bone fractures (20). Therefore, we hypothesize that the positive association between abdominal obesity (reflected by elevated trunk FMR) and fragility fractures may be mediated, in part, by chronic low-grade inflammation associated with visceral adipose tissue. However, it should be carefully noted that BIA utilized in our study cannot directly distinguish visceral from subcutaneous fat. Consequently, the direct linkage between trunk FMR, visceral adiposity, and these specific inflammatory mechanisms remains speculative.

Crucially, the contrasting associations observed—where higher leg FMR was protective while trunk and arm FMR were detrimental—provide supportive evidence for our hypothesis. This site-specific divergence suggests that the anatomical distribution of FMR conveys critical risk information, which may be obscured by aggregate metrics like BMI or total FMR (9). Biomechanically, additional adipose tissue overlying the proximal femur could potentially provide a “cushioning effect” during falls (9). Conversely, trunk and arm adiposity offers no such structural advantage and is frequently associated with adverse metabolic profiles. This heterogeneity aligns with the distinct metabolic characteristics of different fat depots; for instance, gluteofemoral subcutaneous fat (a major component of leg FMR) is generally less pro-inflammatory than the visceral adipose tissue that often correlates with higher trunk FMR (32). These findings imply that the mutual cancellation of opposing site-specific effects may explain the lack of significance in whole-body measurements.

Sarcopenia and fracture are prevalent characteristics of aging as muscle and bone are strongly interconnected. Regarding the decreased muscle mass, an UK Biobank cohort-based study also demonstrated that the fracture and MOF risks were higher in both presarcopenic and sarcopenic participants compared with nonsarcopenic participants, which is consistent to our main finding (21). A meta-analysis including 27,990 participants also suggested that there was significant association between sarcopenia and fracture before and after eliminating confounding factors (22). It was reported that individuals with sarcopenia exhibited a 19% higher risk of falling (23), and the strong correlation between sarcopenia and falls was identified as the most important direct cause of fracture (24). Beyond mechanical factors, sarcopenia is linked to systemic inflammation, hormonal imbalance, and metabolic abnormalities, which can directly or indirectly affect bone health and integrity and consequently increase fracture risk (25).

Regarding the subgroup analysis, we found that the positive associations of arm and trunk FMRs with MOF risk were more pronounced in men than in women. Similarly, prior research has reported a greater risk of hip fracture in men compared with women at high BMI levels after adjusting for femoral neck BMD, highlighting the sex-dependent distribution of adipose tissue (9). Typically, adipose tissue in men is more characterized by visceral abdominal deposition, whereas women tend to exhibit higher subcutaneous fat around the hips rather than the abdomen (26). In females, excess adipose tissue is often associated with increased estrogen activity, which may exert protective effects on the musculoskeletal system (27). Conversely, obesity in males might lead to a concomitant reduction in sex steroids, potentially increasing fracture risk by impairing muscle health in addition to reducing BMD (28). Thus, we propose that these distinct hormonal and fat distribution profiles represent potential mechanisms responsible for the sex-dependent patterns observed in the relationship between site-specific FMR and fragility fractures.

Our study has several limitations. First, while BIA is practical for large-scale epidemiological research, it cannot directly distinguish between visceral and subcutaneous adipose tissue, unlike gold-standard imaging such as CT or MRI. Although prior studies have shown reasonable agreement between 8-electrode BIA and DXA for total fat mass (29–31), the inability to partition trunk fat into specific depots means our mechanistic interpretations regarding metabolic pathways should be viewed as hypothesis-generating. Second, we relied on baseline FMR measurements, which may change over time. Third, although we adjusted for BMI, we did not perform a head-to-head comparison of predictive accuracy; thus, the clinical utility of FMR as a screening tool requires further validation. Fourth, although we adjusted for major covariates, potential residual confounding from unmeasured factors cannot be entirely ruled out (e.g., specific medication use). Finally, as the study population was predominantly of European ancestry, the generalizability of our findings to other ethnic groups remains to be established.

## Conclusion

Our findings indicate that elevated FMR in the arms and trunk is associated with an increased risk of MOF and OF, independent of sex, BMI, and BMD. While further studies are needed to formally compare its predictive performance with traditional metrics like BMI, region-specific FMRs may serve as a potential complementary tool for assessing fracture risk in the elderly.

## Data Availability

The data used in this study were obtained from the UK Biobank (Application Number 896333), a controlled-access resource. The data are not publicly available for unrestricted release. Qualified researchers may apply for access through the UK Biobank access procedure (https://www.ukbiobank.ac.uk/enable-your-research/apply-for-access).
