# Supplementary material for: Associations between total and regional fat-to-muscle mass ratio and fracture risk in elderly population: a prospective cohort study in UK Biobank
Source: Front Med (Lausanne). 2026 Jun 24;13:1830114. doi: 10.3389/fmed.2026.1830114 (PMC13341519; doi:10.3389/fmed.2026.1830114)
Supplement: Supplementary file 1 [file Table_1.docx]

# Table S1. Code selection for incident fractures

| **Fracture location** | **Self-reported codes** | **ICD10 codes** |
| --- | --- | --- |
| Clavicle / collar bone | 1631 | - Fracture of clavicle: S420, S4200, S4201 |
| Shoulder / scapula | 1632 | - Shoulder stress fracture, not elsewhere classified: M8431 |
|  |  | - Fracture of scapula: S421, S4210, S4211 |
|  |  | - Multiple fracture of clavicle, scapula and humerus: S427, S4270, S4271 |
|  |  | - Fracture of other parts of shoulder and upper arm: S428, S4280, S4281 |
|  |  | - Fracture of shoulder girdle, part unspecified: S429, S4290, S4291 |
| Upper arm / humerus / elbow* | 1633 | - Upper arm stress fracture, not elsewhere classified: M8432 |
|  |  | - Fracture of upper end of humerus: S422, S4220, S4221 |
|  |  | - Fracture of shaft of humerus: S423, S4230, S4231 |
|  |  | - Fracture of lower end of humerus: S424, S4240, S4241 |
| Forearm / wrist / Colles fractures* | 1634, 1637 | - Fracture of lower end of radius: S525, S5250, S5251 |
|  |  | - Fracture of lower end of both ulna and radius: S526, S5260, S5261 |
| Radius and ulna, excluding wrist | 1635, 1636 | - Forearm stress fracture, not elsewhere classified: M8433 |
|  |  | - Fracture of upper end of ulna: S520, S5200, S5201 |
|  |  | - Fracture of upper end of radius: S521, S5210, S5211 |
|  |  | - Fracture of shaft of ulna: S522, S5220, S5221 |
|  |  | - Fracture of shaft of radius: S523, S5230, S5231 |
|  |  | - Fracture of shafts of both radius and ulna: S524, S5240, S5241 |
|  |  | - Multiple fractures of forearm: S527, S5270, S5271 |
|  |  | - Fractures of other parts of forearm: S528, S5280, S5281 |
|  |  | - Fractures of forearm, part unspecified: S529, S5290, S5291 |
| Rib | 1644 | - Fracture of rib: S223, S2230, S2231 |
|  |  | - Multiple fractures of ribs: S224, S2240, S2241 |
|  |  | - Fracture of other parts of bony thorax: S228, S2280, S2281, S229, S2290, S2291 |
| Sternum | 1645 | - Fracture of sternum: S222, S2220, S2221 |
| Vertebra / crush fracture / vertebral collapse* | 1646 | - Fatigue fracture of vertebra: M484, M4840, M4844, M4845, M4846, M4847, M4849 |
|  |  | - Collapsed vertebra, not elsewhere classified: M485, M4850, M4854, M4855, M4856, M4857, M4859 |
|  |  | - Fracture of thoracic vertebra: S220, S2200, S2201 |
|  |  | - Multiple fracture of thoracic spine: S221, S2210, S2211 |
|  |  | - Fracture of lumbar vertebra: S320, S3200, S3201 |
|  |  | - Multiple fractures of lumbar spine and pelvis: S327, S3270, S3271 |
|  |  | - Fractures of other and unspecified parts of lumbar spine and pelvis: S328, S3280, S3281 |
| Pelvis | 1647 | - Pelvis stress fracture, not elsewhere classified: M8435 |
|  |  | - Fracture of sacrum: S321, S3210, S3211 |
|  |  | - Fatigue fracture of vertebra (Sacral and sacrococcygeal region): M4848 |
|  |  | - Collapsed vertebra, not elsewhere classified (Sacral and sacrococcygeal region): M4858 |
|  |  | - Fracture of coccyx: S322, S3220, S3221  - Fracture of ilium: S323, S3230, S3231 |
|  |  | - Fracture of acetabulum: S324, S3240, S3241  - Fracture of pubis: S325, S3250, S3251 |
| Neck of femur / hip* | 1648 | - Fracture of neck of femur: S720, S7200, S7201 |
|  |  | - Per-trochanteric fracture: S721, S7210, S7211 |
|  |  | - Sub-trochanteric fracture: S722, S7220, S7221 |
| Shaft of femur | 1649 | - Fracture of shaft of femur: S723, 7230, S7231 |
|  |  | - Multiple fractures of femur: S727, S7270, S7271 |
|  |  | - Fractures of other parts of femur: S728, S7280, S7281 |
|  |  | - Fracture of femur, part unspecified: S729, S7290, S7291 |
| Patella / knee | 1650 | - Fracture of lower end of femur: S724, 7240, S7241 |
|  |  | - Fracture of patella: S820, S8200, S8201 |
|  |  | - Fracture of upper end of tibia: S821, S8210, S8211 |
| Lower leg / ankle | 1651 | - Ankle and foot stress fracture, not elsewhere classified: M8437 |
|  |  | - Fracture lower end of tibia: S823, S8230, S8231 |
|  |  | - Fracture of medial malleolus: S825, S8250, S8251 |
|  |  | - Fracture of lateral malleolus: S826, S8260, S8261 |
| Tibia, fibula | 1652, 1653 | - Lower leg stress fracture, not elsewhere classified: M8436 |
|  |  | - Fracture of shaft of tibia: S822, S8220, S8221 |
|  |  | - Fracture of fibula alone: S824, S8240, S8241 |
|  |  | - Multiple fractures of lower leg: S827, S8270, S8271 |
|  |  | - Fractures of other parts of lower leg: S828, S8280, S8281 |
|  |  | - Maisonneuve's fracture: S8286, S82860, S82861 |
|  |  | - Fracture of lower leg, part unspecified: S829, S8290, S8291 |

* included in MOF location

Abbreviations: **MOF**, major osteoporotic fractures

# Table S2. Baseline characteristics by quintiles of FMR for whole body

| **Characteristic** | **Q1 (n=42377)** | **Q2 (n=42376)** | **Q3 (n=42376)** | **Q4 (n=42376)** | **Q5 (n=42376)** |
| --- | --- | --- | --- | --- | --- |
| **Age (years)** | 64.04 ± 2.85 | 64.18 ± 2.86 | 64.18 ± 2.85 | 64.14 ± 2.85 | 64.11 ± 2.85 |
| **Female (%)** | 3583 (8.5) | 8655 (20.4) | 21739 (51.3) | 36593 (86.4) | 41582 (98.1) |
| **White (%)** | 41031 (97.3) | 40941 (97.1) | 40997 (97.2) | 41058 (97.3) | 40686 (96.4) |
| **Townsend Deprivation Index (%)** | |  |  |  |  |
| 1^st^ | 9994 (23.6) | 9560 (22.6) | 9006 (21.3) | 9155 (21.6) | 7945 (18.8) |
| 2 | 9590 (22.7) | 9417 (22.2) | 9191 (21.7) | 9056 (21.4) | 8461 (20.0) |
| 3 | 8689 (20.5) | 8821 (20.8) | 8852 (20.9) | 8930 (21.1) | 8679 (20.5) |
| 4 | 7666 (18.1) | 7708 (18.2) | 7971 (18.8) | 8111 (19.2) | 8583 (20.3) |
| 5th | 6393 (15.1) | 6839 (16.2) | 7322 (17.3) | 7096 (16.8) | 8668 (20.5) |
| **Higher education (%)** | 28306 (68.0) | 25588 (61.6) | 23199 (55.9) | 21507 (51.8) | 19531 (47.2) |
| **Smoking status (%)** |  |  |  |  |  |
| Never | 20997 (49.8) | 18720 (44.4) | 19966 (47.4) | 22810 (54.2) | 22907 (54.4) |
| Former | 4405 (10.4) | 3886 (9.2) | 3491 (8.3) | 2952 (7.0) | 2553 (6.1) |
| Current | 16790 (39.8) | 19525 (46.3) | 18661 (44.3) | 16322 (38.8) | 16632 (39.5) |
| **Physical activities (MET-minutes/week)** | 3175.80 ± 2906.67 | 2893.70 ± 2784.61 | 2739.06 ± 2629.64 | 2682.82 ± 2535.30 | 2302.02 ± 2308.66 |
| **Alcohol intake frequency (%)** | |  |  |  |  |
| Never or occasional | 9063 (21.4) | 9423 (22.3) | 11835 (28.0) | 14952 (35.3) | 19478 (46.1) |
| 1-2 times a week | 9493 (22.4) | 9895 (23.4) | 10272 (24.3) | 10599 (25.1) | 10312 (24.4) |
| 3-4 times a week | 10743 (25.4) | 10720 (25.3) | 9685 (22.9) | 8765 (20.7) | 6812 (16.1) |
| Daily or almost daily | 13022 (30.8) | 12263 (29.0) | 10506 (24.8) | 7982 (18.9) | 5684 (13.4) |
| **Serum Vitamin D (nmol/L)** | 54.10 [39.00, 68.80] | 51.30 [36.60, 65.90] | 50.00 [35.60, 64.80] | 49.40 [35.20, 63.50] | 44.20 [31.20, 58.50] |
| **BMI (kg/m²)** | 24.55 ± 2.68 | 26.69 ± 3.28 | 27.26 ± 4.24 | 27.57 ± 3.90 | 31.98 ± 4.68 |
| **BMI Categories (%)** |  |  |  |  |  |
| Underweight | 650 (1.5) | 95 (0.2) | 9 (0.0) | 1 (0.0) | 0 (0.0) |
| Normal weight | 22385 (52.8) | 11459 (27.0) | 16324 (38.5) | 10204 (24.1) | 686 (1.6) |
| Overweight | 18568 (43.8) | 24560 (58.0) | 13412 (31.6) | 24142 (57.0) | 15695 (37.0) |
| Obesity | 774 (1.8) | 6262 (14.8) | 12631 (29.8) | 8029 (18.9) | 25995 (61.3) |
| **eBMD T-scores** | -0.22 ± 1.25 | -0.26 ± 1.29 | -0.49 ± 1.31 | -0.67 ± 1.16 | -0.61 ± 1.10 |
| **Glucocorticoids use (%)** | 518 (1.2) | 576 (1.4) | 616 (1.5) | 610 (1.4) | 778 (1.8) |
| **Beef Intake Frequency (%)** | |  |  |  |  |
| Never or less than once a week | 23986 (56.9) | 22323 (52.9) | 22833 (54.1) | 23788 (56.4) | 22962 (54.6) |
| Once a week | 13634 (32.3) | 14563 (34.5) | 13921 (33.0) | 13347 (31.7) | 13754 (32.7) |
| 2 times a week or more | 4571 (10.8) | 5304 (12.6) | 5419 (12.8) | 5016 (11.9) | 5360 (12.7) |
| **Processed Meat Intake (%)** | |  |  |  |  |
| Never or less than once a week | 14288 (33.8) | 14164 (33.5) | 16884 (39.9) | 19907 (47.1) | 19138 (45.3) |
| Once a week | 12606 (29.8) | 12929 (30.6) | 12344 (29.2) | 12375 (29.3) | 12983 (30.7) |
| 2 times a week or more | 15376 (36.4) | 15152 (35.9) | 13039 (30.8) | 9961 (23.6) | 10103 (23.9) |
| **Falls in the last year (%)** | |  |  |  |  |
| 0 | 35675 (84.4) | 34999 (82.8) | 33398 (79.1) | 31592 (74.8) | 29325 (69.5) |
| 1 | 4602 (10.9) | 5187 (12.3) | 6109 (14.5) | 7426 (17.6) | 8322 (19.7) |
| ≥2 | 1997 (4.7) | 2069 (4.9) | 2725 (6.5) | 3220 (7.6) | 4557 (10.8) |
| **Fractured or broken bones in last 5 years (%)** | 3093 (7.3) | 3124 (7.4) | 4047 (9.6) | 4896 (11.6) | 5421 (12.9) |
| **Fractures resulting from simple falls (%)** | 1602 (52.6) | 1824 (59.3) | 2765 (69.1) | 3673 (76.0) | 4269 (79.7) |
| **Rheumatoid arthritis history (%)** | 396 (0.9) | 510 (1.2) | 715 (1.7) | 819 (1.9) | 1052 (2.5) |
| **Diabetes history (%)** | 2593 (6.1) | 4049 (9.6) | 4449 (10.5) | 3777 (8.9) | 5021 (11.8) |

Data are presented as mean ± SD, median [IQR], or n (%).

Abbreviations: **FMR**, fat-to-muscle mass ratio; **eBMD**, estimated bone mineral density; **BMI**, body mass index; **SD**, standard deviation; **IQR**, interquartile range; **MET**, metabolic equivalent of task.

# Table S3. Baseline characteristics in the global population stratified by sex

| **Variable** | **Women** | | **P** | **Men** | | **P** |
| --- | --- | --- | --- | --- | --- | --- |
|  | **No-OF** | **OF** |  | **No-OF** | **OF** |  |
|  | **(n= 99,475)** | **(n=12,677)** |  | **(n= 93,813)** | **(n=5,916)** |  |
| **Age (years)** | 63.97 ± 2.83 | 64.51 ± 2.93 | <0.01* | 64.22 ± 2.86 | 64.69 ± 2.84 | <0.01* |
| **White (%)** | 96,088 (96.9) | 12,432 (98.5) | <0.01* | 90,436 (96.9) | 5,757 (97.9) | <0.01* |
| **Townsend Deprivation Index (%)** | | | | | |  |
| 1st | 21,272 (21.4) | 2,541 (20.1) | <0.01* | 20,724 (22.1) | 1,123 (19.0) | <0.01* |
| 2 | 21,446 (21.6) | 2,633 (20.8) | | 20,486 (21.9) | 1,150 (19.4) | |
| 3 | 20,869 (21.0) | 2,611 (20.6) | | 19,328 (20.6) | 1,163 (19.7) | |
| 4 | 19,141 (19.3) | 2,548 (20.1) | | 17,225 (18.4) | 1,125 (19.0) | |
| 5th | 16,663 (16.8) | 2,336 (18.4) | | 15,967 (17.0) | 1,352 (22.9) | |
| **Higher education (%)** | 50,956 (52.3) | 6,453 (52.2) | 0.87 | 57,326 (62.4) | 3,396 (59.0) | <0.01* |
| **Smoking status (%)** | | | |  |  |  |
| Never | 56,911 (57.5) | 6,988 (55.4) | <0.01* | 39,257 (42.1) | 2,244 (38.2) | <0.01* |
| Former | 35,494 (35.9) | 4,624 (36.7) | | 44,965 (48.2) | 2,847 (48.5) | |
| Current | 6,500 (6.6) | 1,001 (7.9) | | 9,008 (9.7) | 778 (13.3) | |
| **Physical activities (MET-minutes/week)** | 2733.39 ± 2537.74 | 2694.09 ± 2509.92 | 0.17 | 2829.89 ± 2792.94 | 2839.71 ± 2890.34 | 0.82 |
| **Alcohol intake frequency (%)** | | | | |  |  |
| Never or occasional | 38,784 (39.1) | 4,932 (39.0) | <0.01* | 19,665 (21.0) | 1,370 (23.2) | <0.01* |
| 1-2 times a week | 23,885 (24.1) | 2,937 (23.2) | | 22,446 (24.0) | 1,303 (22.1) | |
| 3-4 times a week | 18,884 (19.0) | 2,366 (18.7) | | 24,099 (25.7) | 1,376 (23.3) | |
| Daily or almost daily | 17,751 (17.9) | 2,415 (19.1) | | 27,444 (29.3) | 1,847 (31.3) | |
| **Serum Vitamin D (nmol/L)** | 49.30 [35.10, 63.90] | 49.50 [34.90, 64.70] | 0.33 | 50.50 [35.90, 65.20] | 47.90 [32.60, 63.50] | <0.01* |
| **BMI (kg/m²)** | 27.40 ± 4.89 | 27.02 ± 4.88 | <0.01* | 27.90 ± 4.04 | 27.76 ± 4.32 | 0.01* |
| **BMI Categories (%)** | | | |  |  |  |
| Underweight | 533 (0.5) | 105 (0.8) | <0.01* | 103 (0.1) | 14 (0.2) | <0.01* |
| Normal weight | 33,386 (33.6) | 4,711 (37.2) | | 21,377 (22.8) | 1,584 (26.8) | |
| Overweight | 40,593 (40.8) | 4,937 (38.9) | | 48,101 (51.3) | 2,746 (46.4) | |
| Obesity | 24,963 (25.1) | 2,924 (23.1) | | 24,232 (25.8) | 1,572 (26.6) | |
| **eBMD T-scores** | -0.73 ± 1.05 | -1.13 ± 0.98 | <0.01* | -0.06 ± 1.33 | -0.52 ± 1.29 | <0.01* |
| **Glucocorticoids use (%)** | 1,368 (1.4) | 308 (2.4) | <0.01* | 1,312 (1.4) | 110 (1.9) | <0.01* |
| **Beef intake frequency (%)** | | | | |  |  |
| Never or less than once a week | 57,957 (58.6) | 7,330 (58.2) | 0.02* | 47,629 (51.0) | 2,976 (50.6) | 0.01* |
| Once a week | 30,039 (30.4) | 3,762 (29.9) | | 33,369 (35.7) | 2,049 (34.8) | |
| 2 times a week or more | 10,964 (11.1) | 1,502 (11.9) | | 12,345 (13.2) | 859 (14.6) | |
| **Processed meat intake (%)** | | | | |  |  |
| Never or less than once a week | 50,576 (51.0) | 6,370 (50.4) | 0.5 | 25,841 (27.6) | 1,594 (27.0) | 0.02* |
| Once a week | 28,755 (29.0) | 3,707 (29.4) | | 29,022 (31.0) | 1,753 (29.7) | |
| 2 times a week or more | 19,866 (20.0) | 2,553 (20.2) | | 38,666 (41.3) | 2,546 (43.2) | |
| **Falls in the last year (%)** | | | | |  |  |
| 0 | 74,043 (74.7) | 8,484 (67.2) | <0.01* | 78,018 (83.4) | 4,444 (75.4) | <0.01* |
| 1 | 17,513 (17.7) | 2,641 (20.9) | | 10,619 (11.4) | 873 (14.8) | |
| ≥2 | 7,607 (7.7) | 1,506 (11.9) | | 4,879 (5.2) | 576 (9.8) | |
| **Fractured or broken bones in last 5 years(%)** | 11,441 (11.6) | 2,457 (19.5) | <0.01* | 5,935 (6.4) | 748 (12.7) | <0.01* |
| **Fractures resulting from simple falls (%)** | 8,650 (76.5) | 1,878 (77.4) | 0.35 | 3,156 (54.1) | 449 (61.2) | <0.01* |
| **Rheumatoid arthritis history (%)** | 1,951 (2.0) | 378 (3.0) | <0.01* | 1,060 (1.1) | 103 (1.7) | <0.01* |
| **Diabetes history (%)** | 7,067 (7.1) | 1,086 (8.6) | <0.01* | 10,891 (11.6) | 845 (14.3) | <0.01* |
| **History of bilateraloophorectomy (%)** | 11,013 (11.3) | 1,318 (10.7) | 0.03* | - | - | - |
| **Hormone replacement therapy_history(%)** | 57,797 (58.3) | 7,512 (59.5) | 0.01* | - | - | - |

Data are presented as mean ± SD, median [IQR], or n (%).

Abbreviations: **OF**, osteoporotic fractures; **eBMD**, estimated bone mineral density; **BMI**, body mass index; **SD**, standard deviation; **IQR**, interquartile range; **MET**, metabolic equivalent of task.

* P < 0.05 was considered statistically significant.

# Table S4. Associations between FMR indices and OF, stratified by sex

| **FMR** | **HR** | **Model 3** | **P** | **HR** | **Model 3** | **P** | **P interaction** |
| --- | --- | --- | --- | --- | --- | --- | --- |
|  |  | **(95% CI)** |  |  | **(95% CI)** |  |  |
|  | **Women** | | | **Men** | | |  |
| **Whole body** |  |  |  |  |  |  | 0.01** |
| Q2 | 0.96 | (0.90, 1.02) | 0.22 | 0.89 | (0.81, 0.98) | 0.02 |  |
| Q3 | 1.03 | (0.95, 1.11) | 0.50 | 0.92 | (0.83, 1.02) | 0.09 |  |
| Q4 | 1.03 | (0.95, 1.13) | 0.44 | 0.94 | (0.84, 1.05) | 0.26 |  |
| Q5 | 1.16 | (1.05, 1.28) | <0.01* | 1.05 | (0.93, 1.19) | 0.43 |  |
| **Trunk** |  |  |  |  |  |  | 0.08 |
| Q2 | 0.99 | (0.92, 1.05) | 0.69 | 0.88 | (0.80, 0.96) | <0.01* |  |
| Q3 | 1.06 | (0.99, 1.13) | 0.12 | 0.9 | (0.82, 1.00) | 0.05 |  |
| Q4 | 1.09 | (1.01, 1.18) | 0.03* | 0.99 | (0.89, 1.11) | 0.92 |  |
| Q5 | 1.18 | (1.08, 1.29) | <0.01* | 1.07 | (0.95, 1.21) | 0.26 |  |
| **Arms** |  |  |  |  |  |  | 0.01** |
| Q2 | 0.99 | (0.93, 1.06) | 0.87 | 0.97 | (0.88, 1.07) | 0.56 |  |
| Q3 | 1.06 | (0.97, 1.15) | 0.21 | 1 | (0.90, 1.12) | 0.94 |  |
| Q4 | 1.13 | (1.03, 1.25) | <0.01* | 1.11 | (0.99, 1.25) | 0.07 |  |
| Q5 | 1.19 | (1.05, 1.35) | <0.01* | 1.28 | (1.12, 1.45) | <0.01* |  |
| **Legs** |  |  |  |  |  |  | 0.01** |
| Q2 | 0.93 | (0.87, 0.99) | 0.02* | 0.9 | (0.82, 0.98) | 0.02* |  |
| Q3 | 0.93 | (0.87, 1.01) | 0.09 | 0.86 | (0.78, 0.96) | <0.01* |  |
| Q4 | 0.94 | (0.86, 1.03) | 0.17 | 0.87 | (0.78, 0.97) | <0.01* |  |
| Q5 | 1 | (0.89, 1.11) | 0.93 | 0.91 | (0.81, 1.02) | 0.11 |  |

Model 3 was adjusted for age, gender, race, Townsend deprivation index, education, smoking status, alcohol intake frequency, beef intake, processed meat intake, serum vitamin D, glucocorticoid use, falls in the last year, prior fractures, diabetes, and rheumatoid arthritis, BMI categories and eBMD T-scores.

Abbreviations: **FMR**, fat-to-muscle mass ratio; **OF**, osteoporotic fractures **eBMD**, estimated bone mineral density; **BMI**, body mass index; **HR**, hazard ratio; **CI**, confidence interval.

* P < 0.05 was considered statistically significant.

** P for interaction < 0.05 was considered statistically significant in the multiplicative interaction.

# Table S5. Associations between FMR indices and OF and OF in women, with additional adjustment.

| **FMR** | **HR** | **Model**  **(95% CI)** | **P** | **HR** | **Model**  **(95% CI)** | **P** |
| --- | --- | --- | --- | --- | --- | --- |
|  | **MOF** | | | **OF** | | |
| **Whole body** |  |  |  |  |  |  |
| Q2 | 0.95 | (0.88, 1.03) | 0.25 | 0.96 | (0.90, 1.03) | 0.26 |
| Q3 | 1.01 | (0.93, 1.11) | 0.75 | 1.03 | (0.95, 1.11) | 0.51 |
| Q4 | 1.01 | (0.91, 1.11) | 0.91 | 1.03 | (0.95, 1.12) | 0.49 |
| Q5 | 1.08 | (0.96, 1.22) | 0.20 | 1.16 | (1.05, 1.28) | < 0.01* |
| **Trunk** |  |  |  |  |  |  |
| Q2 | 1.01 | (0.94, 1.09) | 0.78 | 1 | (0.93, 1.06) | 0.9 |
| Q3 | 1.07 | (0.99, 1.17) | 0.11 | 1.06 | (0.99, 1.14) | 0.11 |
| Q4 | 1.08 | (0.98, 1.19) | 0.11 | 1.09 | (1.01, 1.18) | 0.03* |
| Q5 | 1.15 | (1.04, 1.28) | 0.01* | 1.19 | (1.09, 1.29) | < 0.01* |
| **Arms** |  |  |  |  |  |  |
| Q2 | 1.01 | (0.93, 1.09) | 0.77 | 1 | (0.93, 1.07) | 0.92 |
| Q3 | 1.07 | (0.97, 1.18) | 0.19 | 1.05 | (0.96, 1.14) | 0.27 |
| Q4 | 1.15 | (1.02, 1.29) | 0.02* | 1.13 | (1.02, 1.24) | 0.01* |
| Q5 | 1.23 | (1.06, 1.43) | < 0.01* | 1.19 | (1.05, 1.35) | < 0.01* |
| **Legs** |  |  |  |  |  |  |
| Q2 | 0.91 | (0.84, 0.98) | 0.01* | 0.92 | (0.86, 0.99) | 0.02* |
| Q3 | 0.89 | (0.81, 0.98) | 0.01* | 0.92 | (0.85, 1.00) | 0.04* |
| Q4 | 0.85 | (0.77, 0.95) | < 0.01* | 0.93 | (0.85, 1.01) | 0.09 |
| Q5 | 0.9 | (0.79, 1.03) | 0.13 | 0.99 | (0.89, 1.11) | 0.88 |

Model was adjusted for age, race, Townsend deprivation index, education, smoking status, alcohol intake frequency, beef intake frequency, processed meat intake, serum vitamin D, glucocorticoids use at baseline, falls in the last year, fractured or broken bones in last 5 years, diabetes history, rheumatoid arthritis history, BMI categories, eBMD T-scores, history of bilateral oophorectomy and hormone replacement therapy (HRT) use.

Abbreviations: **FMR**, fat-to-muscle mass ratio; **OF,** osteoporotic fractures; **MOF**, major osteoporotic fractures; **eBMD**, estimated bone mineral density; **BMI**, body mass index; **HR**, hazard ratio; **CI**, confidence interval; **HRT**, hormone replacement therapy.

* P < 0.05 was considered statistically significant.

# Table S6. Collinearity Diagnostics for Regional FMR Models

| Region | Variables | GVIF | Df | Adjusted GVIF |
| --- | --- | --- | --- | --- |
| Whole body | FMR_Whole | 4.2275 | 4 | 1.1975 |
|  | BMI_categories | 2.5548 | 3 | 1.1692 |
|  | eBMD_T_scores | 1.0968 | 1 | 1.0473 |
|  |  |  |  |  |
| Trunk | FMR_Trunk | 2.3698 | 4 | 1.1139 |
|  | BMI_categories | 2.1532 | 3 | 1.1364 |
|  | eBMD_T_scores | 1.0946 | 1 | 1.0462 |
|  |  |  |  |  |
| Arms | FMR_Arms | 5.8412 | 4 | 1.2468 |
|  | BMI_categories | 3.2155 | 3 | 1.2149 |
|  | eBMD_T_scores | 1.0954 | 1 | 1.0466 |
|  |  |  |  |  |
| Legs | FMR_Legs | 12.3832 | 4 | 1.3696 |
|  | BMI_categories | 2.8488 | 3 | 1.1906 |
|  | eBMD_T_scores | 1.1017 | 1 | 1.0496 |

Multicollinearity was evaluated using the Generalized Variance Inflation Factor (GVIF), and the “Standardized VIF” was calculated to adjust for varying degrees of freedom across categorical and continuous variables. A Standardized VIF value below 2.0 (equivalent to a standard VIF < 4.0) indicates no significant multicollinearity.

Beyond the core variables displayed, all models were adjusted for age, race, gender, Townsend Deprivation Index, education, smoking status, alcohol intake frequency, serum vitamin D, glucocorticoids use, beef and processed meat intake, and history of fractures, falls, rheumatoid arthritis, and diabete.

Abbreviations: **FMR**, fat-to-muscle ratio; **BMI**, body mass index; **eBMD**, estimated bone mineral density; **GVIF**, generalized variance inflation factor; **Df**, degrees of freedom.

# **Figure S1.** Cox proportional hazard regression analyses on associations between FMR indices and risk of incident OF.


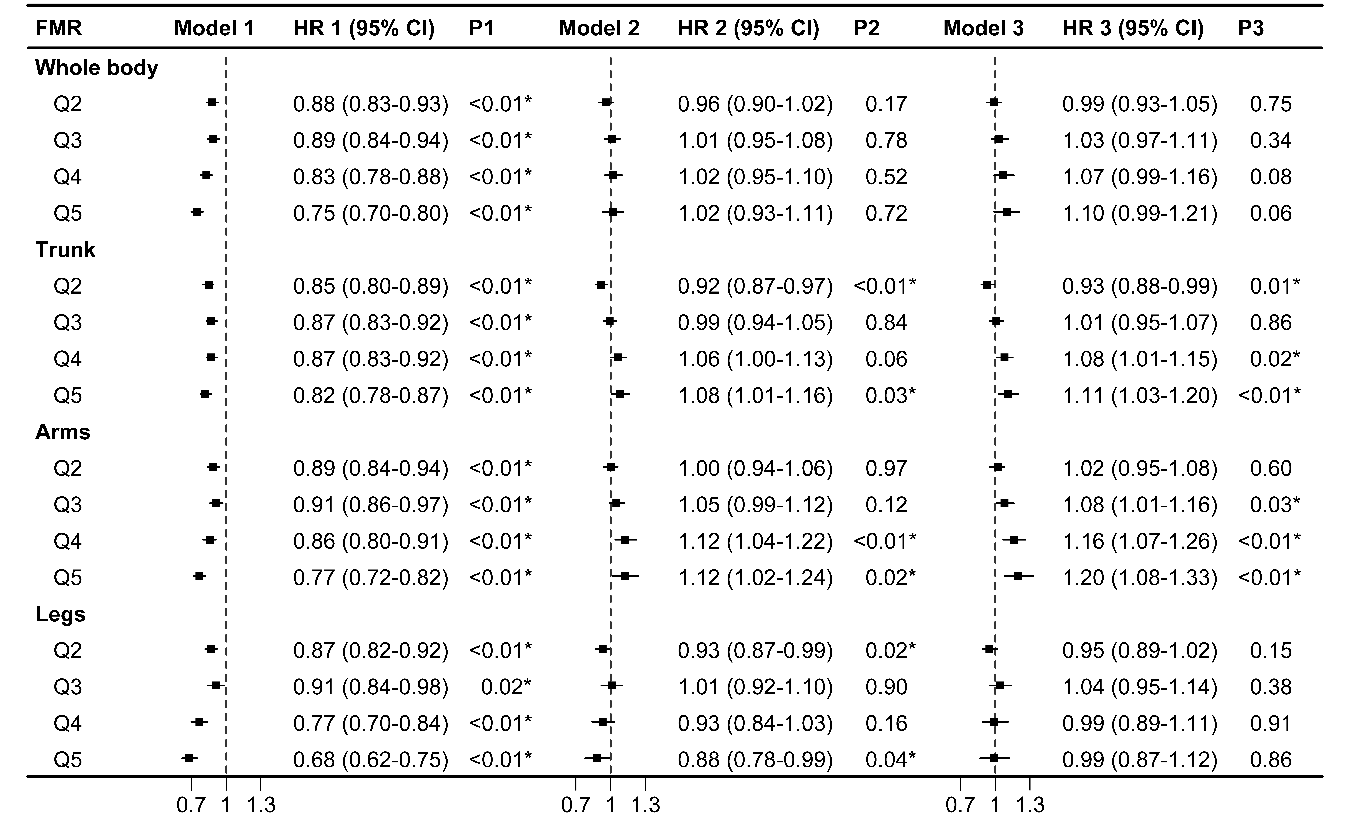


Note: The forest plots illustrate the HRs and 95% CIs for the associations between FMR indices (Whole body, Trunk, Arms, and Legs) and the risk of incident OF. Model 1 was adjusted for age, gender, race, Townsend deprivation index, education, smoking status, alcohol intake frequency, beef intake, processed meat intake, serum vitamin D, glucocorticoid use, falls in the last year, prior fractures, diabetes, and rheumatoid arthritis. Model 2 was further adjusted for BMI categories. Model 3 was further adjusted for both BMI categories and eBMD T-scores. “*” indicates statistical significance with P < 0.05.

Abbreviations: **FMR**, fat-to-muscle mass ratio; **OF**, osteoporotic fractures; **eBMD**, estimated bone mineral density; **BMI**, body mass index ; **HR**, hazard ratio; **CI**, confidence interval.

# Figure S2. Forest plots of HRs and 95% CI for the risk of OF by FMR, stratified by sex.


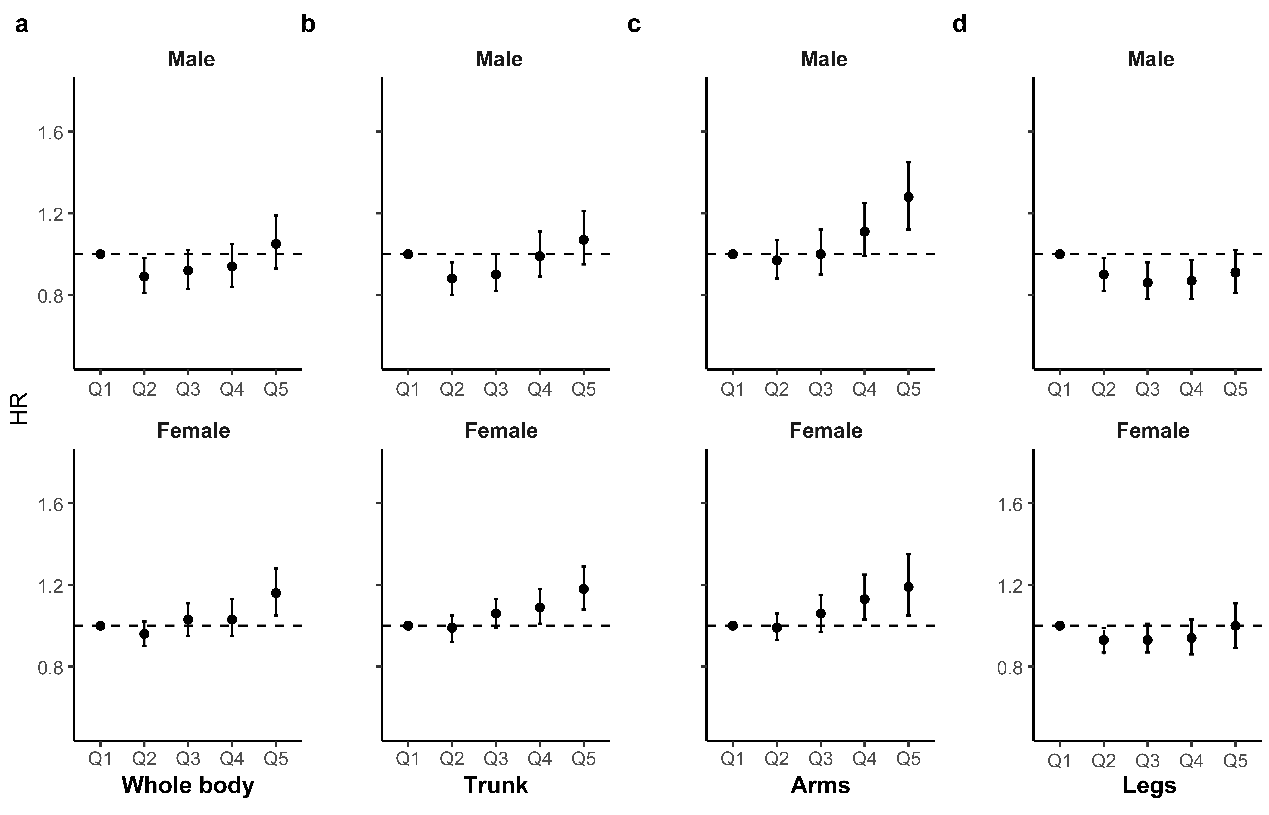


Note: The forest plots show the HRs and 95% CIs for the associations between FMR indices and the risk of MOF, stratified by Male and Female. Participants were categorized into quintiles (Q1-Q5), with Q1 as the reference group. The model was adjusted for age, race, Townsend deprivation index, education, smoking status, alcohol intake frequency, beef and processed meat intake, serum vitamin D, glucocorticoid use, falls in the last year, prior fractures, diabetes, rheumatoid arthritis, BMI categories, and eBMD T-scores.

Abbreviations: **FMR**, fat-to-muscle mass ratio; **MOF**, major osteoporotic fractures **eBMD**, estimated bone mineral density; **BMI**, body mass index; **HR**, hazard ratio; **CI**, confidence interval.

#
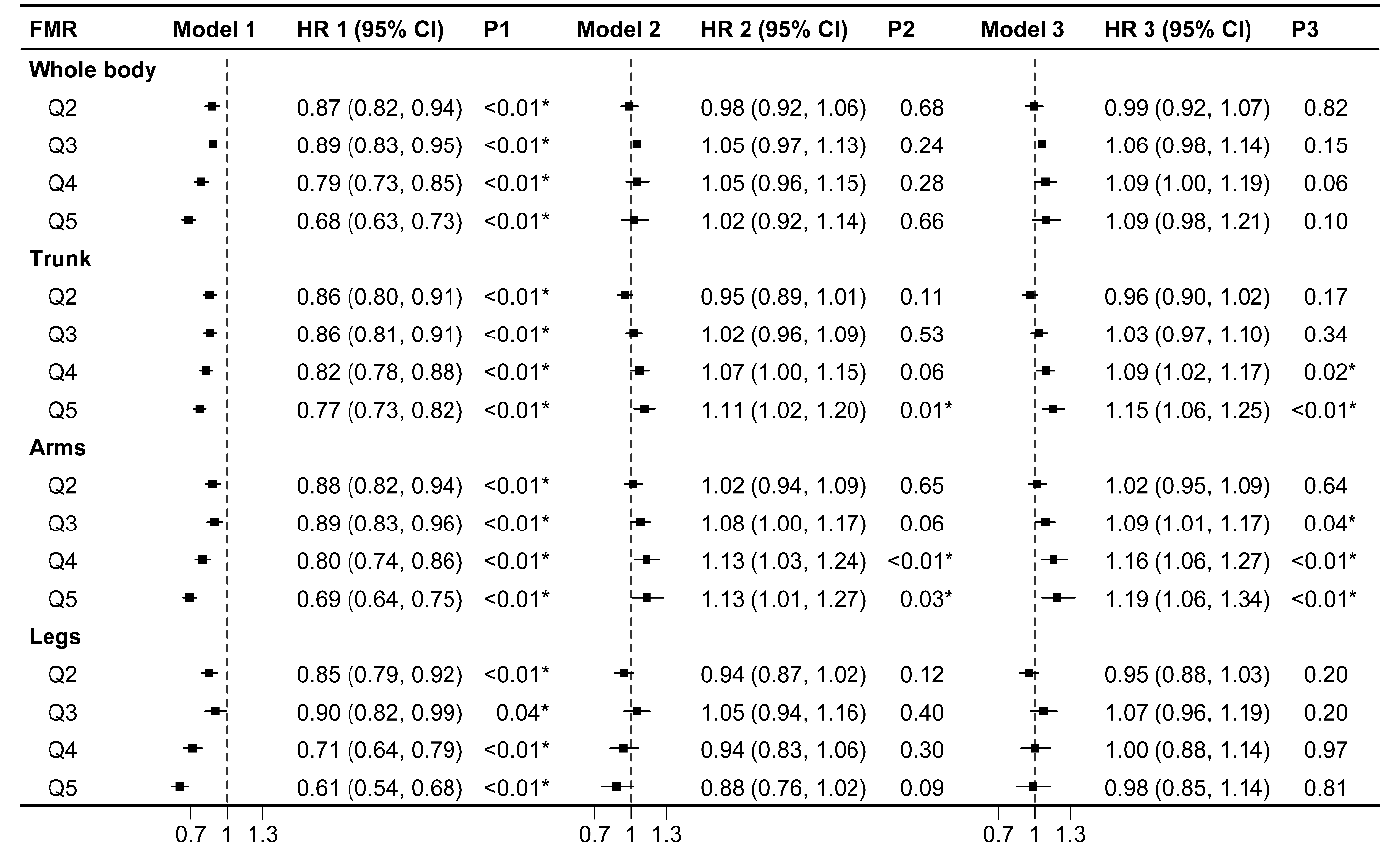
Figure S3. Associations between FMR indices and MOF based on multiple imputation data.

Note: The forest plots illustrate the HRs and 95% CIs for the associations between FMR indices (Whole body, Trunk, Arms, and Legs) and the risk of incident MOF based on multiple imputation data. Model 1 was adjusted for age, gender, race, Townsend deprivation index, education, smoking status, alcohol intake frequency, beef intake, processed meat intake, serum vitamin D, glucocorticoid use, falls in the last year, prior fractures, diabetes, and rheumatoid arthritis. Model 2 was further adjusted for BMI categories. Model 3 was further adjusted for both BMI categories and eBMD T-scores. “*” indicates statistical significance with P < 0.05.

Abbreviations: **FMR**, fat-to-muscle mass ratio; **MOF**, osteoporotic fractures; **eBMD**, estimated bone mineral density; **BMI**, body mass index ; **HR**, hazard ratio; **CI**, confidence interval.

# Figure S4. Associations between FMR indices and OF based on multiple imputation data.


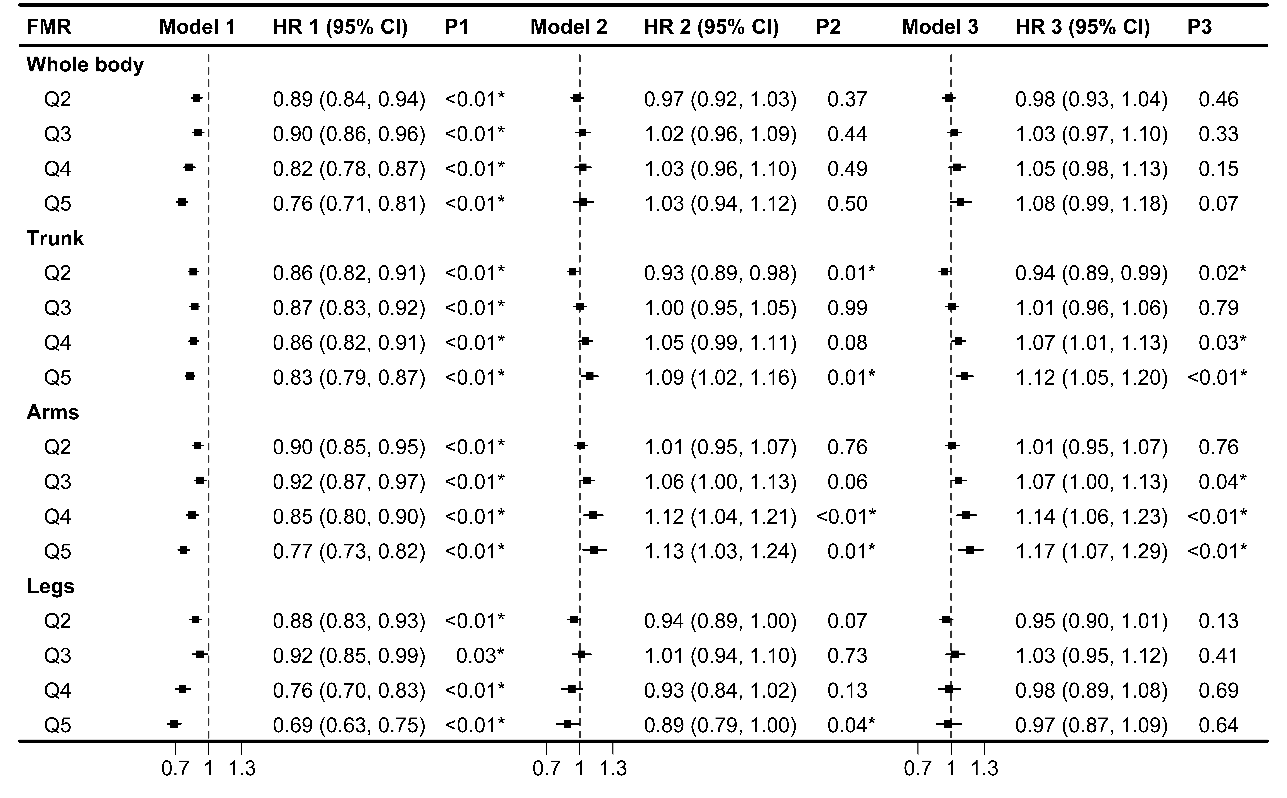


Note:The forest plots illustrate the HRs and 95% CIs for the associations between FMR indices (Whole body, Trunk, Arms, and Legs) and the risk of incident OF based on multiple imputation data. Model 1 was adjusted for age, gender, race, Townsend deprivation index, education, smoking status, alcohol intake frequency, beef intake, processed meat intake, serum vitamin D, glucocorticoid use, falls in the last year, prior fractures, diabetes, and rheumatoid arthritis. Model 2 was further adjusted for BMI categories. Model 3 was further adjusted for both BMI categories and eBMD T-scores. “*” indicates statistical significance with P < 0.05.

Abbreviations: **FMR**, fat-to-muscle mass ratio; **OF**, osteoporotic fractures; **eBMD**, estimated bone mineral density; **BMI**, body mass index ; **HR**, hazard ratio; **CI**, confidence interval.

# Figure S5. Associations between FMR indices and MOF after excluding the first two years of incident cases.


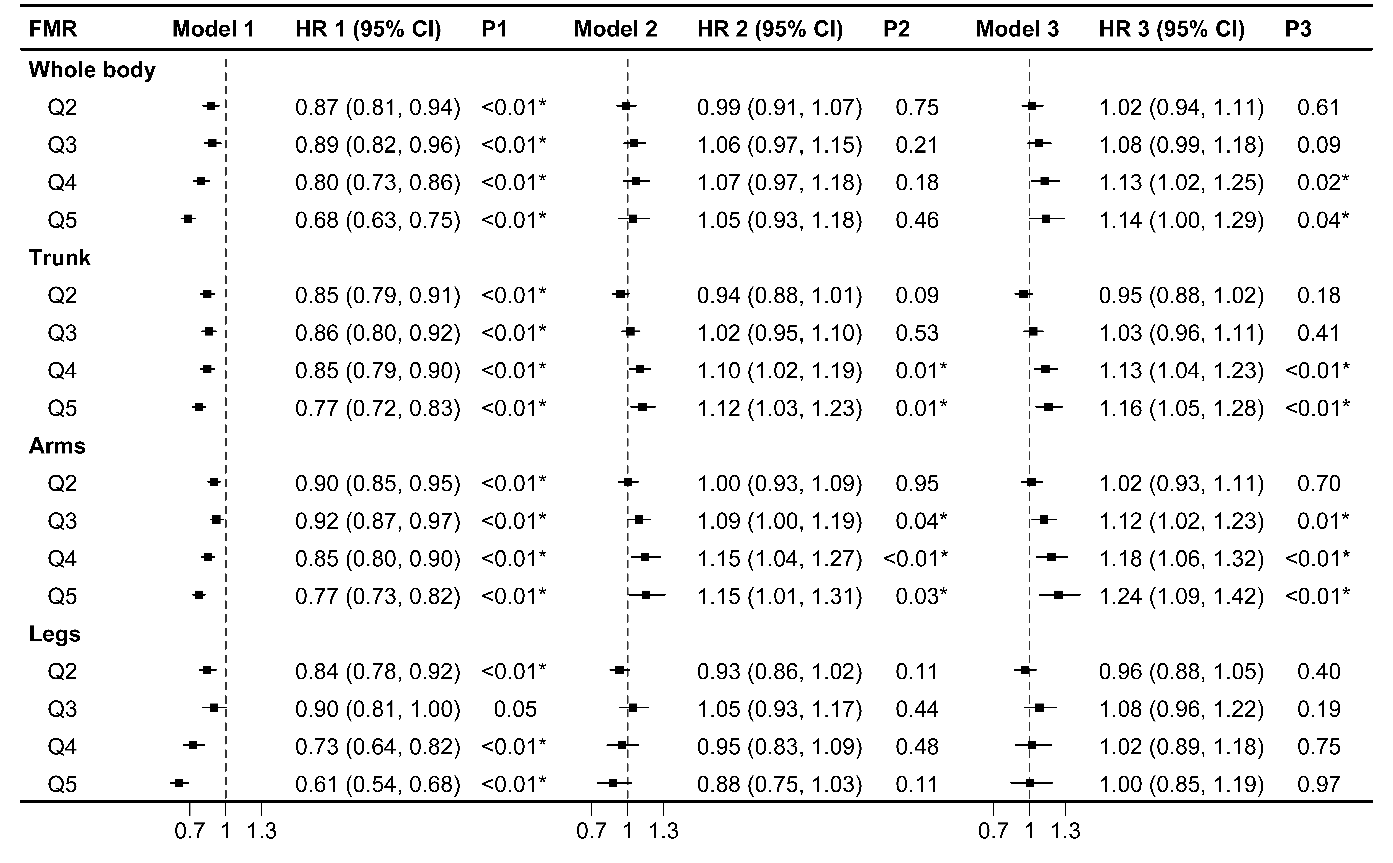


Note:The forest plots illustrate the HRs and 95% CIs for the associations between FMR indices (Whole body, Trunk, Arms, and Legs) and the risk of incident MOF after excluding the first two years of incident cases. Model 1 was adjusted for age, gender, race, Townsend deprivation index, education, smoking status, alcohol intake frequency, beef intake, processed meat intake, serum vitamin D, glucocorticoid use, falls in the last year, prior fractures, diabetes, and rheumatoid arthritis. Model 2 was further adjusted for BMI categories. Model 3 was further adjusted for both BMI categories and eBMD T-scores. “*” indicates statistical significance with P < 0.05.

Abbreviations: **FMR**, fat-to-muscle mass ratio; **MOF**, osteoporotic fractures; **eBMD**, estimated bone mineral density; **BMI**, body mass index ; **HR**, hazard ratio; **CI**, confidence interval.

# Figure S6. Associations between FMR indices and OF risk after excluding the first two years of incident cases.


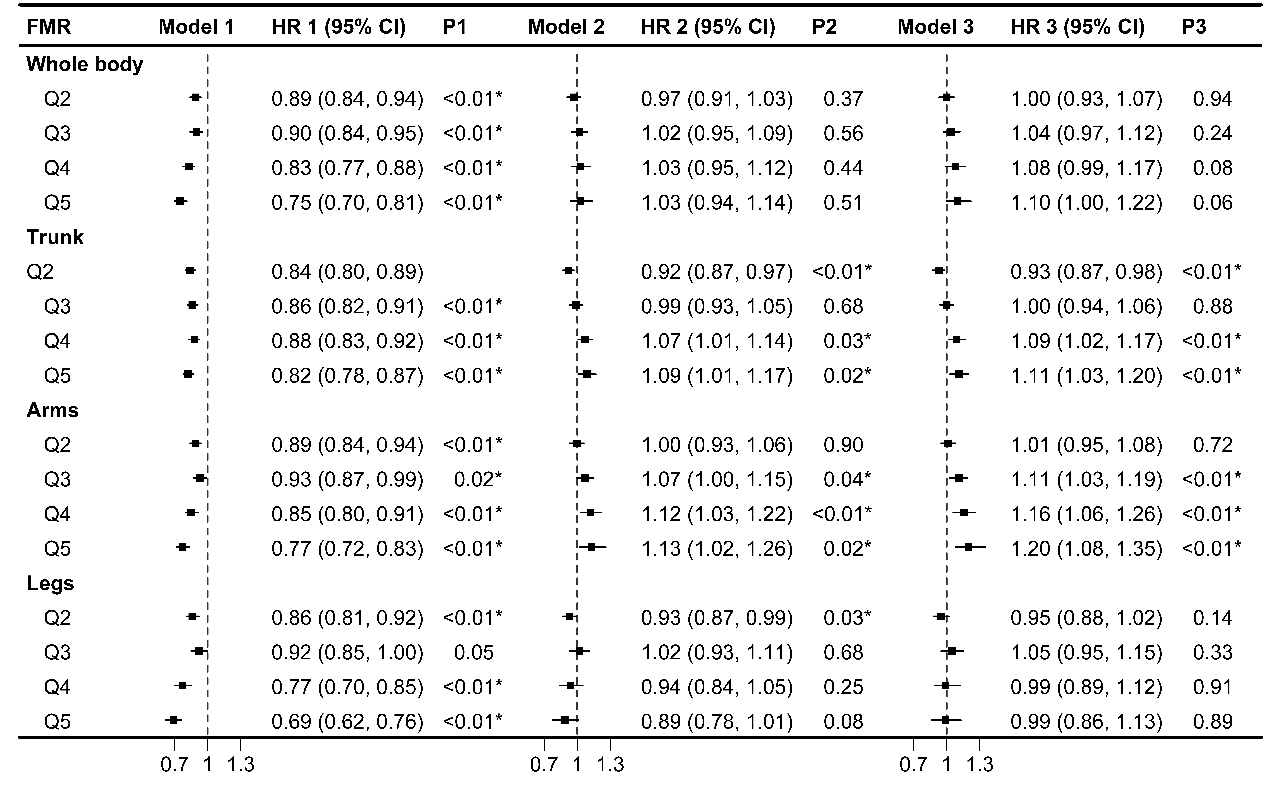


Note: The forest plots illustrate the HRs and 95% CIs for the associations between FMR indices (Whole body, Trunk, Arms, and Legs) and the risk of incident OF after excluding the first two years of incident cases. Model 1 was adjusted for age, gender, race, Townsend deprivation index, education, smoking status, alcohol intake frequency, beef intake, processed meat intake, serum vitamin D, glucocorticoid use, falls in the last year, prior fractures, diabetes, and rheumatoid arthritis. Model 2 was further adjusted for BMI categories. Model 3 was further adjusted for both BMI categories and eBMD T-scores. “*” indicates statistical significance with P < 0.05.

Abbreviations: **FMR**, fat-to-muscle mass ratio; **OF**, osteoporotic fractures; **eBMD**, estimated bone mineral density; **BMI**, body mass index ; **HR**, hazard ratio; **CI**, confidence interval.

# Figure S7. Associations between FMR indices and MOF risk: Competing Risk Model (a), Physical Activity Adjustment (b), and ICD-10 Defined Outcomes (c).


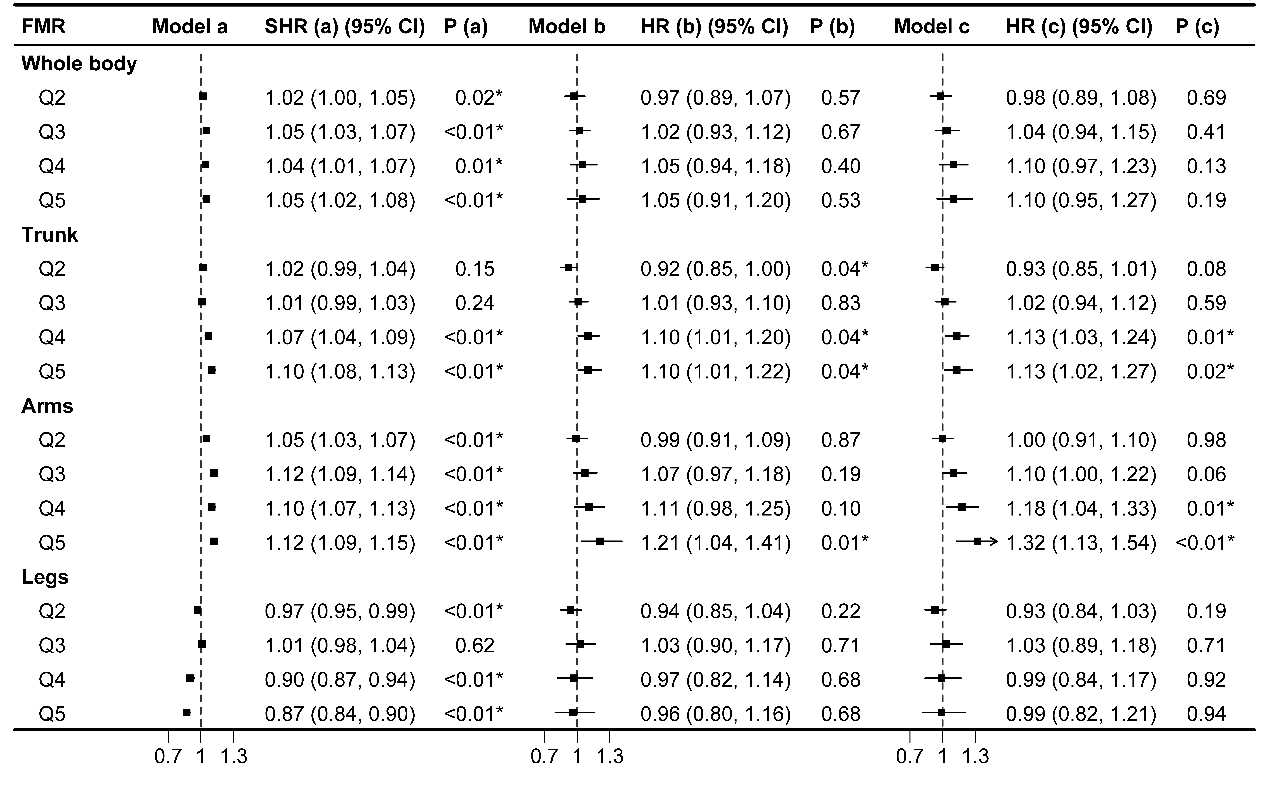


Note: The forest plots illustrate the results of multiple sensitivity analyses for the associations between FMR indices and incident MOF risk: (a) Competing Risk Analysis: Fine-Gray models were employed to account for mortality as a competing event, with results reported as Subdistribution Hazard Ratios (SHR). (b) Physical Activity Adjustment: Associations were further adjusted for physical activity levels in addition to Model 3 covariates. (c) Outcome Definition: Fracture outcomes were restricted solely to ICD-10 hospital records, excluding self-reported data. All models were adjusted for age, gender, race, Townsend deprivation index, education, smoking status, alcohol intake frequency, beef and processed meat intake, serum vitamin D, glucocorticoid use, falls in the last year, prior fractures, diabetes, rheumatoid arthritis, BMI categories and eBMD T-scores. “*” indicates P < 0.05.

**Abbreviations:** **FMR**, fat-to-muscle mass ratio; **MOF**, major osteoporotic fracture; **eBMD**, estimated bone mineral density; **BMI**, body mass index; **SHR**, subdistribution hazard ratio; **HR**, hazard ratio; **CI,** confidence interval; **ICD-10**, International Classification of Diseases 10th Revision.
