# Supplementary material for: Associations between total and regional fat-to-muscle mass ratio and fracture risk in elderly population: a prospective cohort study in UK Biobank
Source: Front Med (Lausanne). 2026 Jun 24;13:1830114. doi: 10.3389/fmed.2026.1830114 (PMC13341519; doi:10.3389/fmed.2026.1830114)
Supplement: Supplementary file 2 [file Data_Sheet_1.pdf]

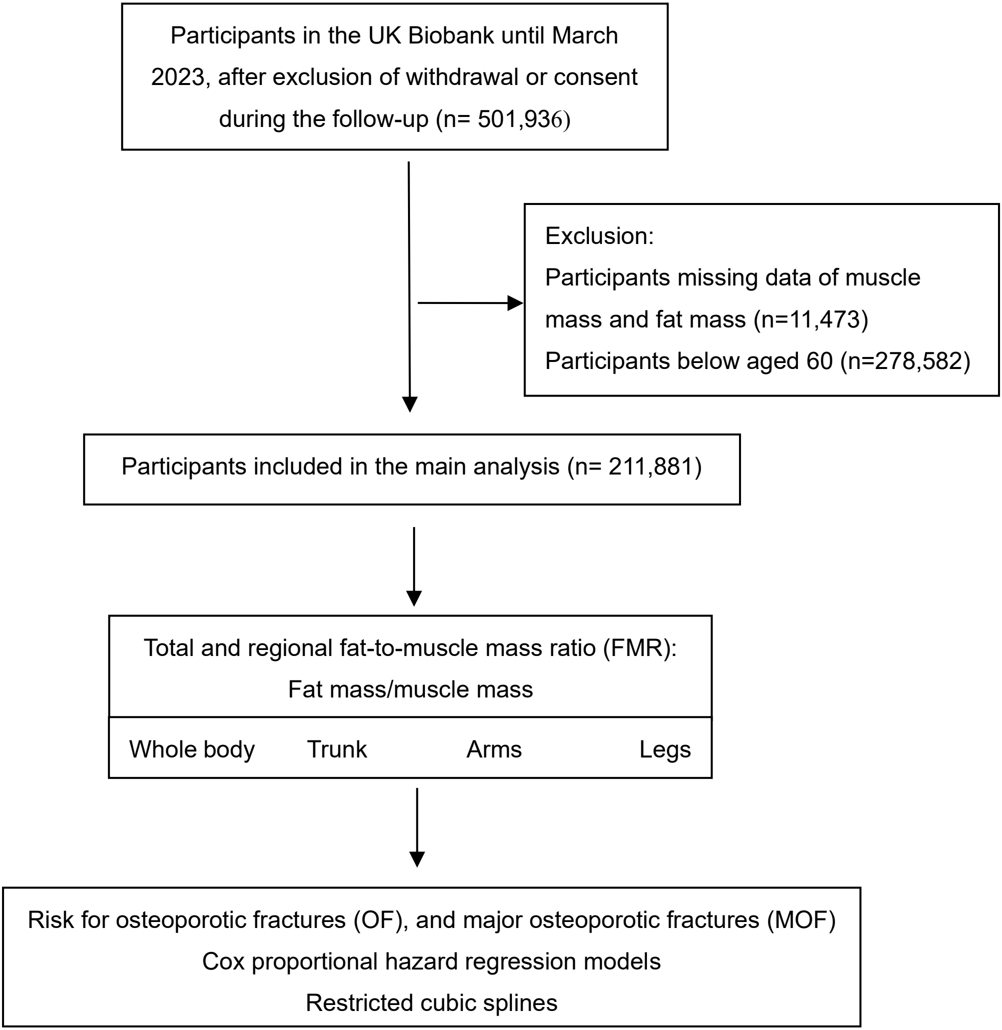
Participants in the UK Biobank until March 2023, after exclusion of withdrawal or consent during the follow-up (n= 501,936)
Exclusion:
Participants missing data of muscle mass and fat mass (n=11,473)
Participants below aged 60 (n=278,582)
Participants included in the main analysis (n= 211,881)
Total and regional fat-to-muscle mass ratio (FMR):
Fat mass/muscle mass
Whole body
Trunk
Arms
Legs
Risk for osteoporotic fractures (OF), and major osteoporotic fractures (MOF)
Cox proportional hazard regression models
Restricted cubic splines
