# Supplementary material for: Associations between total and regional fat-to-muscle mass ratio and fracture risk in elderly population: a prospective cohort study in UK Biobank
Source: Front Med (Lausanne). 2026 Jun 24;13:1830114. doi: 10.3389/fmed.2026.1830114 (PMC13341519; doi:10.3389/fmed.2026.1830114)
Supplement: Supplementary file 3 [file Data_Sheet_2.pdf]

FMR	Model 1	HR 1 (95% CI)	P1	Model 2	HR 2 (95% CI)	P2	Model 3	HR 3 (95% CI)	P3
**Whole body**									
Q2		0.86 (0.80, 0.92)	0.01*		0.97 (0.90-1.04)	0.39		1.01 (0.93-1.09)	0.89
Q3		0.87 (0.81, 0.94)	<0.01*		1.03 (0.95-1.12)	0.41		1.06 (0.97-1.15)	0.19
Q4		0.78 (0.72, 0.85)	<0.01*		1.05 (0.96-1.15)	0.31		1.11 (1.01-1.22)	0.04*
Q5		0.67 (0.62, 0.73)	<0.01*		1.02 (0.91-1.15)	0.69		1.12 (0.99-1.26)	0.06
**Trunk**									
Q2		0.85 (0.79, 0.90)	<0.01*		0.94 (0.88-1.01)	0.08		0.96 (0.89-1.03)	0.20
Q3		0.86 (0.80, 0.91)	<0.01*		1.02 (0.95-1.09)	0.55		1.03 (0.96-1.11)	0.39
Q4		0.83 (0.78, 0.89)	<0.01*		1.08 (1.01-1.17)	0.03*		1.11 (1.03-1.20)	0.01*
Q5		0.77 (0.72, 0.82)	<0.01*		1.12 (1.03-1.22)	0.01*		1.16 (1.06-1.27)	<0.01*
**Arms**									
Q2		0.87 (0.81, 0.94)	<0.01*		1.01 (0.94-1.09)	0.74		1.03 (0.95-1.12)	0.47
Q3		0.88 (0.82, 0.95)	<0.01*		1.07 (0.99-1.16)	0.10		1.09 (1.00-1.19)	0.04*
Q4		0.80 (0.74, 0.86)	<0.01*		1.14 (1.03-1.26)	<0.01*		1.18 (1.06-1.31)	<0.01*
Q5		0.68 (0.63, 0.74)	<0.01*		1.15 (1.02-1.30)	0.03*		1.24 (1.09-1.41)	<0.01*
**Legs**									
Q2		0.84 (0.77, 0.90)	<0.01*		0.92 (0.85-1.00)	0.06		0.96 (0.88-1.04)	0.31
Q3		0.88 (0.80, 0.98)	0.01*		1.03 (0.92-1.15)	0.58		1.08 (0.96-1.21)	0.22
Q4		0.71 (0.63, 0.80)	<0.01*		0.94 (0.82-1.07)	0.36		1.02 (0.89-1.17)	0.76
Q5		0.59 (0.53, 0.67)	<0.01*		0.87 (0.75-1.02)	0.08		1.01 (0.86-1.19)	0.91
