# Supplementary figures and images for: Associations between total and regional fat-to-muscle mass ratio and fracture risk in elderly population: a prospective cohort study in UK Biobank
Source: Front Med (Lausanne). 2026 Jun 24;13:1830114. doi: 10.3389/fmed.2026.1830114 (PMC13341519; doi:10.3389/fmed.2026.1830114)

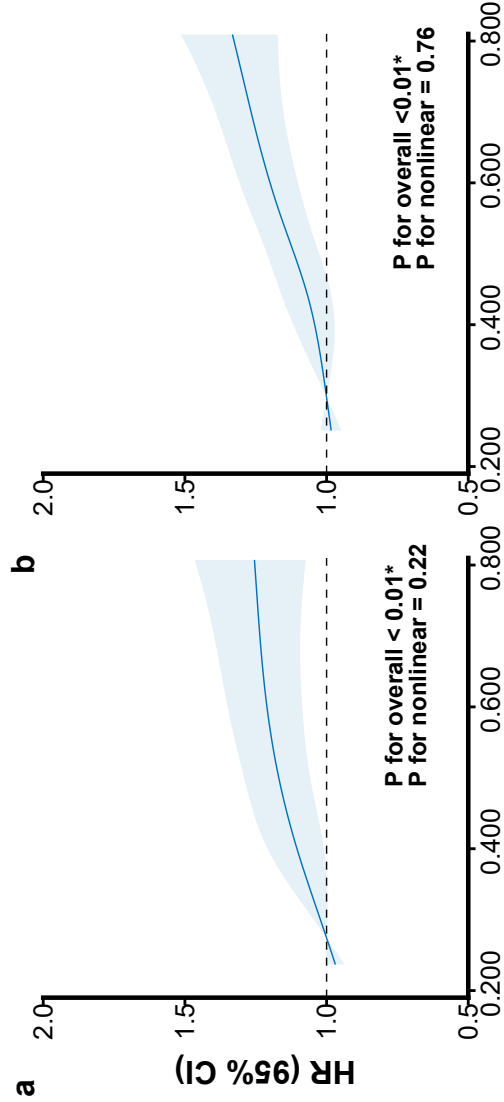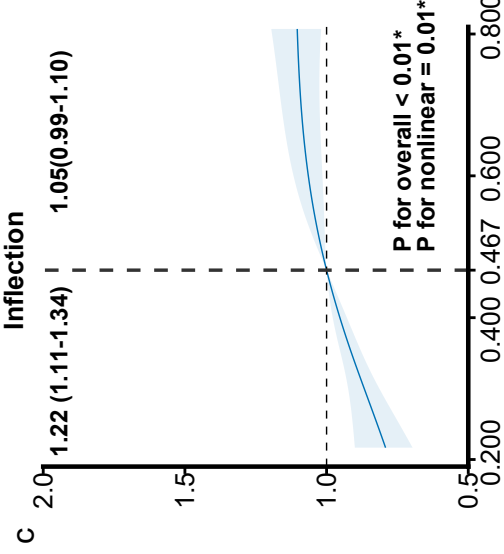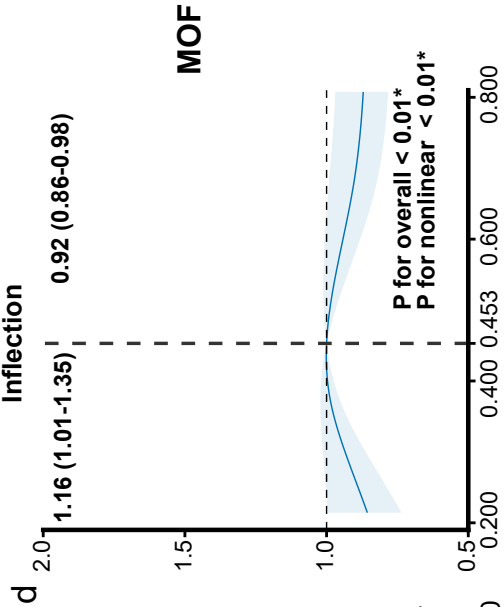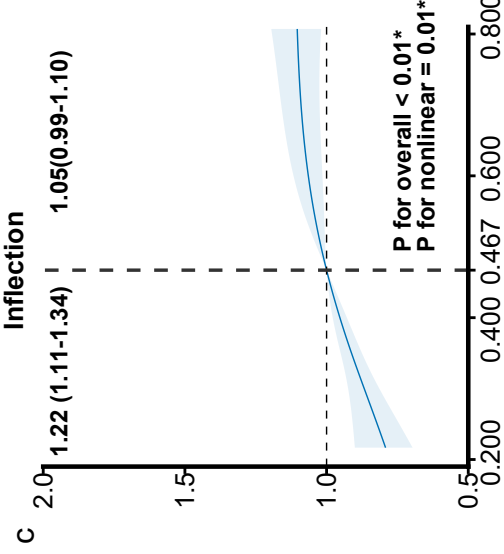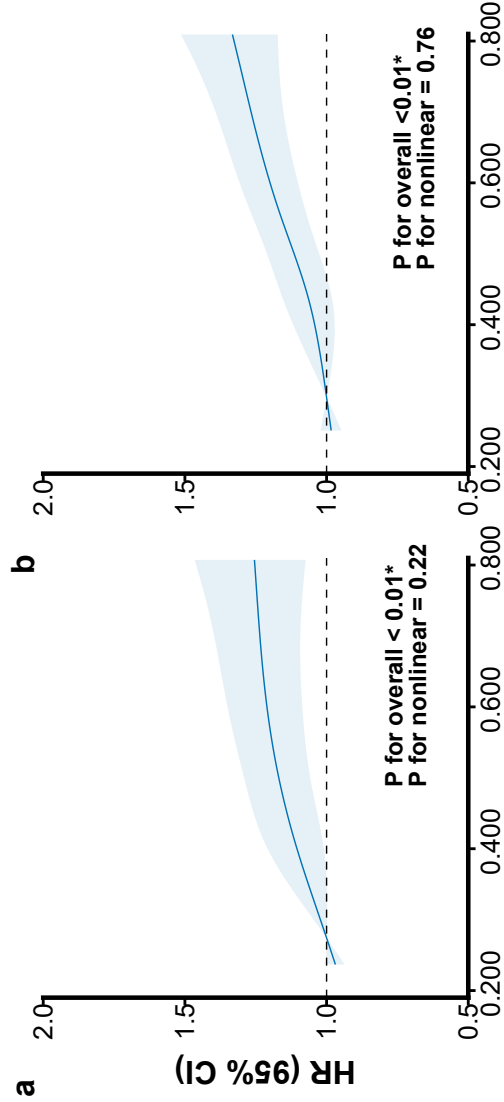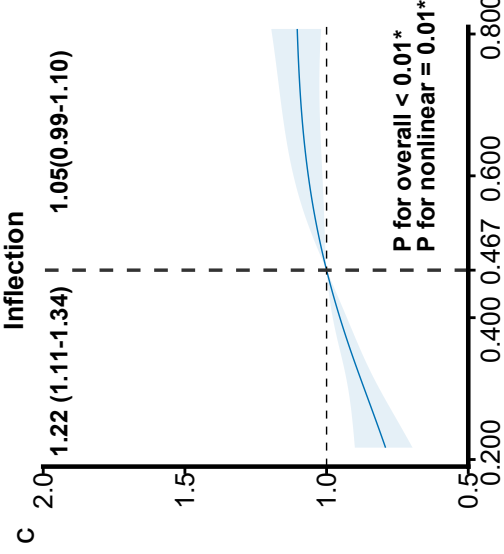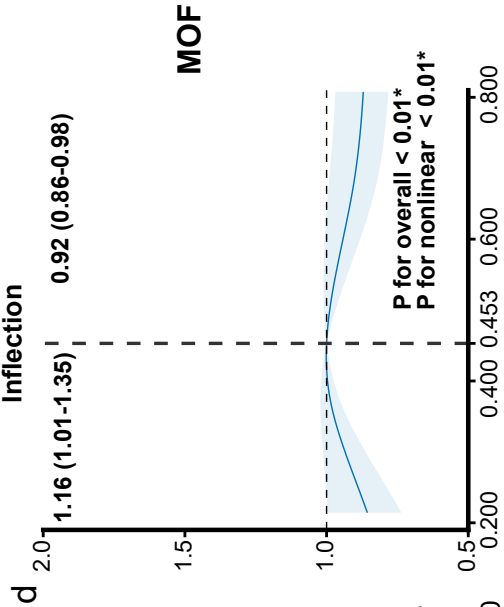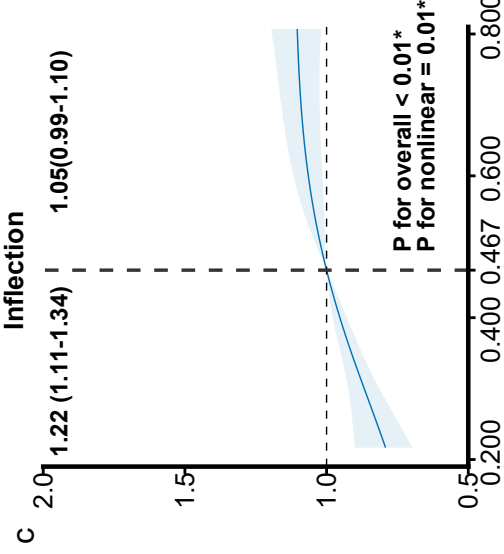

Supplement: Supplementary file 4 [file Data_Sheet_3.pdf]

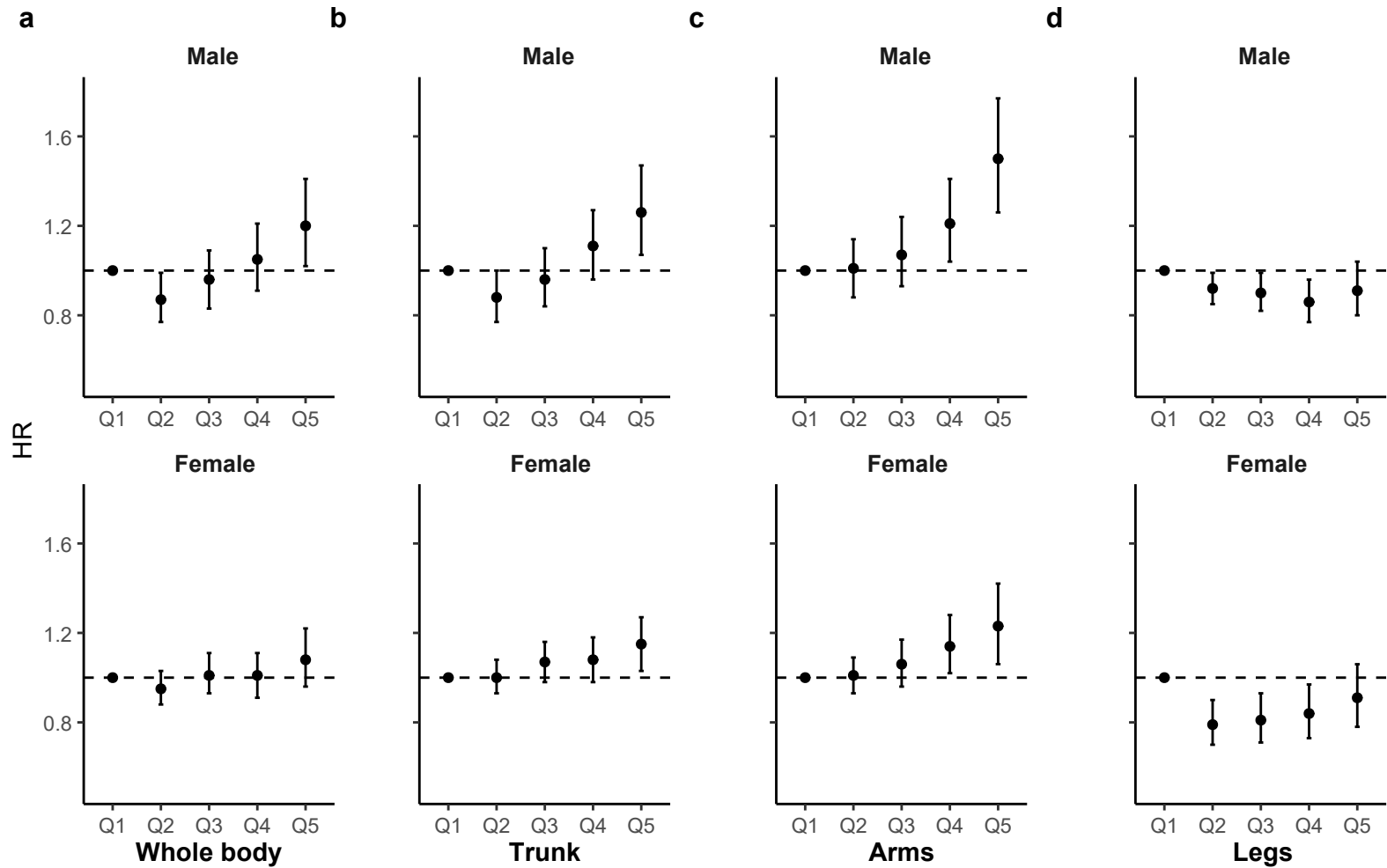

Supplement: Supplementary file 5 [file Data_Sheet_4.pdf]

| FMR               | Model 1                                                                           | HR 1 (95% CI)    | P1     | Model 2                                                                           | HR 2 (95% CI)    | P2     | Model 3                                                                             | HR 3 (95% CI)    | P3     |
|-------------------|-----------------------------------------------------------------------------------|------------------|--------|-----------------------------------------------------------------------------------|------------------|--------|-------------------------------------------------------------------------------------|------------------|--------|
| <b>Whole body</b> |                                                                                   |                  |        |                                                                                   |                  |        |                                                                                     |                  |        |
| Q2                | 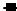 | 0.88 (0.83-0.93) | <0.01* | 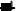 | 0.96 (0.90-1.02) | 0.17   | 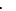 | 0.99 (0.93-1.05) | 0.75   |
| Q3                | 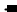 | 0.89 (0.84-0.94) | <0.01* | 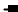 | 1.01 (0.95-1.08) | 0.78   | 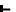 | 1.03 (0.97-1.11) | 0.34   |
| Q4                | 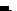 | 0.83 (0.78-0.88) | <0.01* | 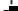 | 1.02 (0.95-1.10) | 0.52   | 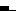 | 1.07 (0.99-1.16) | 0.08   |
| Q5                | 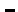 | 0.75 (0.70-0.80) | <0.01* | 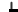 | 1.02 (0.93-1.11) | 0.72   | 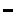 | 1.10 (0.99-1.21) | 0.06   |
| <b>Trunk</b>      |                                                                                   |                  |        |                                                                                   |                  |        |                                                                                     |                  |        |
| Q2                | 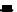 | 0.85 (0.80-0.89) | <0.01* | 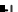 | 0.92 (0.87-0.97) | <0.01* | 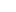 | 0.93 (0.88-0.99) | 0.01*  |
| Q3                | 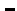 | 0.87 (0.83-0.92) | <0.01* | 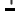 | 0.99 (0.94-1.05) | 0.84   | 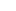 | 1.01 (0.95-1.07) | 0.86   |
| Q4                | 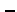 | 0.87 (0.83-0.92) | <0.01* | 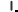 | 1.06 (1.00-1.13) | 0.06   | 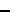 | 1.08 (1.01-1.15) | 0.02*  |
| Q5                | 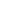 | 0.82 (0.78-0.87) | <0.01* | 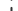 | 1.08 (1.01-1.16) | 0.03*  | 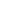 | 1.11 (1.03-1.20) | <0.01* |
| <b>Arms</b>       |                                                                                   |                  |        |                                                                                   |                  |        |                                                                                     |                  |        |
| Q2                | 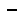 | 0.89 (0.84-0.94) | <0.01* | 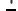 | 1.00 (0.94-1.06) | 0.97   | 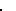 | 1.02 (0.95-1.08) | 0.60   |
| Q3                | 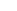 | 0.91 (0.86-0.97) | <0.01* | 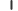 | 1.05 (0.99-1.12) | 0.12   | 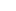 | 1.08 (1.01-1.16) | 0.03*  |
| Q4                | 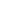 | 0.86 (0.80-0.91) | <0.01* | 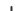 | 1.12 (1.04-1.22) | <0.01* | 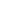 | 1.16 (1.07-1.26) | <0.01* |
| Q5                | 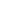 | 0.77 (0.72-0.82) | <0.01* | 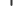 | 1.12 (1.02-1.24) | 0.02*  | 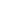 | 1.20 (1.08-1.33) | <0.01* |
| <b>Legs</b>       |                                                                                   |                  |        |                                                                                   |                  |        |                                                                                     |                  |        |
| Q2                | 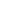 | 0.87 (0.82-0.92) | <0.01* | 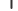 | 0.93 (0.87-0.99) | 0.02*  | 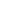 | 0.95 (0.89-1.02) | 0.15   |
| Q3                | 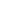 | 0.91 (0.84-0.98) | 0.02*  | 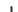 | 1.01 (0.92-1.10) | 0.90   | 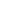 | 1.04 (0.95-1.14) | 0.38   |
| Q4                | 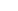 | 0.77 (0.70-0.84) | <0.01* | 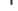 | 0.93 (0.84-1.03) | 0.16   | 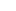 | 0.99 (0.89-1.11) | 0.91   |
| Q5                | 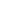 | 0.68 (0.62-0.75) | <0.01* | 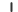 | 0.88 (0.78-0.99) | 0.04*  | 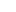 | 0.99 (0.87-1.12) | 0.86   |
|                   | 0.7 1 1.3                                                                         |                  |        | 0.7 1 1.3                                                                         |                  |        | 0.7 1 1.3                                                                           |                  |        |

Supplement: Supplementary file 6 [file Data_Sheet_5.pdf]

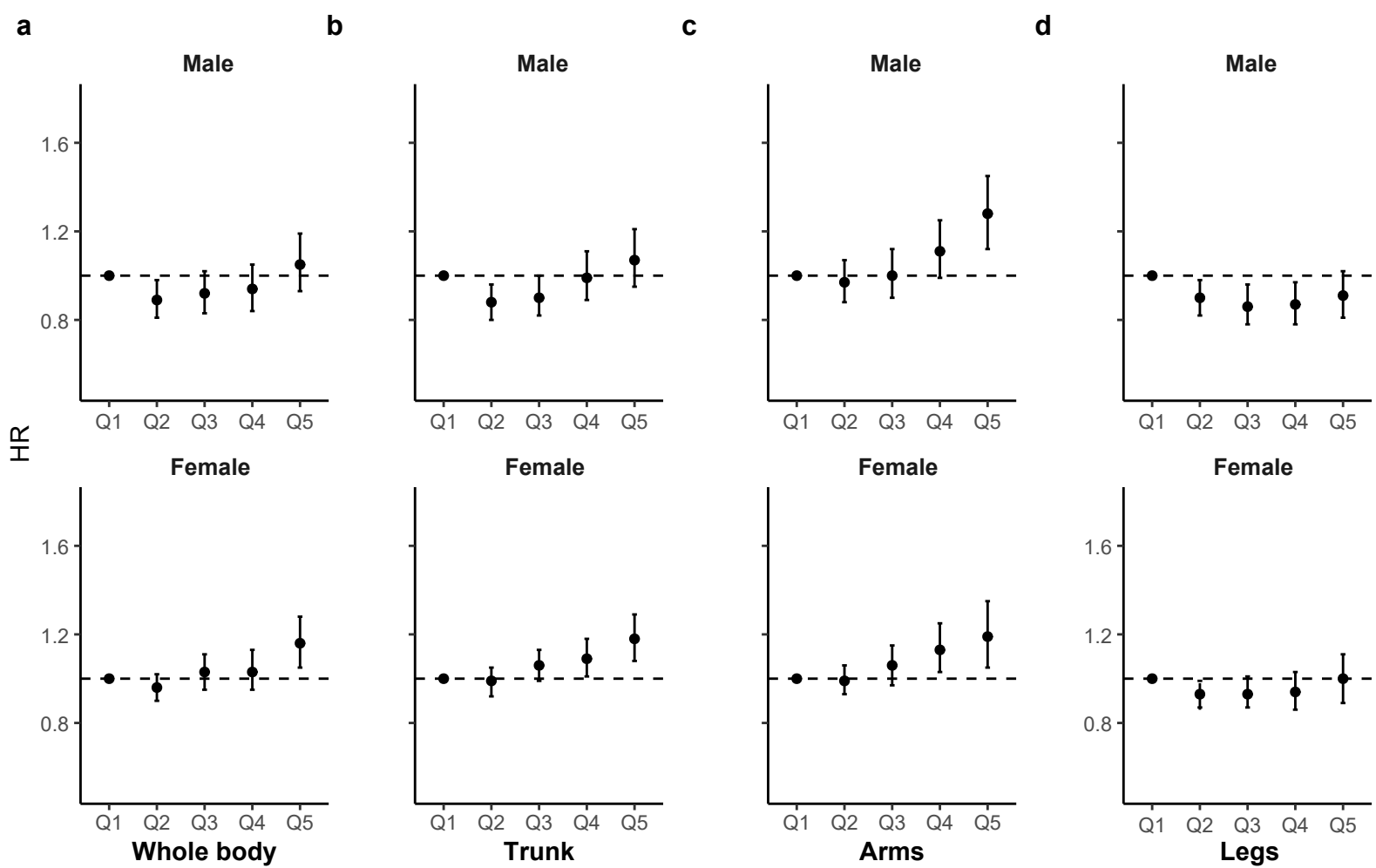

Supplement: Supplementary file 7 [file Data_Sheet_6.pdf]

| FMR               | Model 1                                                                           | HR 1 (95% CI)     | P1     | Model 2                                                                           | HR 2 (95% CI)     | P2     | Model 3                                                                             | HR 3 (95% CI)     | P3     |
|-------------------|-----------------------------------------------------------------------------------|-------------------|--------|-----------------------------------------------------------------------------------|-------------------|--------|-------------------------------------------------------------------------------------|-------------------|--------|
| <b>Whole body</b> |                                                                                   |                   |        |                                                                                   |                   |        |                                                                                     |                   |        |
| Q2                | 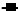 | 0.87 (0.81, 0.94) | <0.01* | 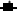 | 0.99 (0.91, 1.07) | 0.75   | 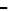 | 1.02 (0.94, 1.11) | 0.61   |
| Q3                | 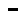 | 0.89 (0.82, 0.96) | <0.01* | 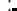 | 1.06 (0.97, 1.15) | 0.21   | 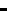 | 1.08 (0.99, 1.18) | 0.09   |
| Q4                | 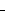 | 0.80 (0.73, 0.86) | <0.01* | 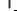 | 1.07 (0.97, 1.18) | 0.18   | 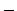 | 1.13 (1.02, 1.25) | 0.02*  |
| Q5                | 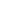 | 0.68 (0.63, 0.75) | <0.01* | 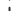 | 1.05 (0.93, 1.18) | 0.46   | 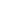 | 1.14 (1.00, 1.29) | 0.04*  |
| <b>Trunk</b>      |                                                                                   |                   |        |                                                                                   |                   |        |                                                                                     |                   |        |
| Q2                | 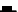 | 0.85 (0.79, 0.91) | <0.01* | 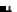 | 0.94 (0.88, 1.01) | 0.09   | 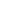 | 0.95 (0.88, 1.02) | 0.18   |
| Q3                | 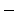 | 0.86 (0.80, 0.92) | <0.01* | 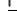 | 1.02 (0.95, 1.10) | 0.53   | 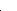 | 1.03 (0.96, 1.11) | 0.41   |
| Q4                | 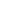 | 0.85 (0.79, 0.90) | <0.01* | 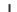 | 1.10 (1.02, 1.19) | 0.01*  | 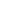 | 1.13 (1.04, 1.23) | <0.01* |
| Q5                | 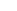 | 0.77 (0.72, 0.83) | <0.01* | 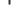 | 1.12 (1.03, 1.23) | 0.01*  | 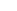 | 1.16 (1.05, 1.28) | <0.01* |
| <b>Arms</b>       |                                                                                   |                   |        |                                                                                   |                   |        |                                                                                     |                   |        |
| Q2                | 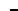 | 0.90 (0.85, 0.95) | <0.01* | 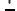 | 1.00 (0.93, 1.09) | 0.95   | 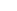 | 1.02 (0.93, 1.11) | 0.70   |
| Q3                | 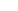 | 0.92 (0.87, 0.97) | <0.01* | 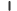 | 1.09 (1.00, 1.19) | 0.04*  | 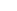 | 1.12 (1.02, 1.23) | 0.01*  |
| Q4                | 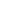 | 0.85 (0.80, 0.90) | <0.01* | 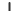 | 1.15 (1.04, 1.27) | <0.01* | 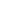 | 1.18 (1.06, 1.32) | <0.01* |
| Q5                | 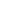 | 0.77 (0.73, 0.82) | <0.01* | 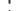 | 1.15 (1.01, 1.31) | 0.03*  | 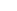 | 1.24 (1.09, 1.42) | <0.01* |
| <b>Legs</b>       |                                                                                   |                   |        |                                                                                   |                   |        |                                                                                     |                   |        |
| Q2                | 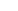 | 0.84 (0.78, 0.92) | <0.01* | 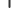 | 0.93 (0.86, 1.02) | 0.11   | 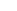 | 0.96 (0.88, 1.05) | 0.40   |
| Q3                | 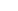 | 0.90 (0.81, 1.00) | 0.05   | 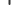 | 1.05 (0.93, 1.17) | 0.44   | 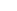 | 1.08 (0.96, 1.22) | 0.19   |
| Q4                | 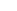 | 0.73 (0.64, 0.82) | <0.01* | 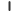 | 0.95 (0.83, 1.09) | 0.48   | 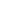 | 1.02 (0.89, 1.18) | 0.75   |
| Q5                | 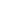 | 0.61 (0.54, 0.68) | <0.01* | 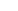 | 0.88 (0.75, 1.03) | 0.11   | 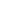 | 1.00 (0.85, 1.19) | 0.97   |
|                   | 0.7 1 1.3                                                                         |                   |        | 0.7 1 1.3                                                                         |                   |        | 0.7 1 1.3                                                                           |                   |        |

Supplement: Supplementary file 10 [file Data_Sheet_9.pdf]

| FMR               | Model 1                                                                           | HR 1 (95% CI)     | P1     | Model 2                                                                           | HR 2 (95% CI)     | P2     | Model 3                                                                             | HR 3 (95% CI)     | P3     |
|-------------------|-----------------------------------------------------------------------------------|-------------------|--------|-----------------------------------------------------------------------------------|-------------------|--------|-------------------------------------------------------------------------------------|-------------------|--------|
| <b>Whole body</b> |                                                                                   |                   |        |                                                                                   |                   |        |                                                                                     |                   |        |
| Q2                | 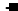 | 0.89 (0.84, 0.94) | <0.01* | 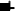 | 0.97 (0.91, 1.03) | 0.37   | 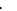 | 1.00 (0.93, 1.07) | 0.94   |
| Q3                | 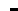 | 0.90 (0.84, 0.95) | <0.01* | 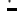 | 1.02 (0.95, 1.09) | 0.56   | 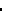 | 1.04 (0.97, 1.12) | 0.24   |
| Q4                | 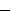 | 0.83 (0.77, 0.88) | <0.01* | 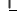 | 1.03 (0.95, 1.12) | 0.44   | 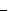 | 1.08 (0.99, 1.17) | 0.08   |
| Q5                | 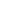 | 0.75 (0.70, 0.81) | <0.01* | 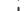 | 1.03 (0.94, 1.14) | 0.51   | 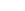 | 1.10 (1.00, 1.22) | 0.06   |
| <b>Trunk</b>      |                                                                                   |                   |        |                                                                                   |                   |        |                                                                                     |                   |        |
| Q2                | 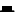 | 0.84 (0.80, 0.89) |        | 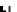 | 0.92 (0.87, 0.97) | <0.01* | 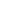 | 0.93 (0.87, 0.98) | <0.01* |
| Q3                | 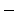 | 0.86 (0.82, 0.91) | <0.01* | 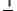 | 0.99 (0.93, 1.05) | 0.68   | 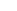 | 1.00 (0.94, 1.06) | 0.88   |
| Q4                | 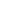 | 0.88 (0.83, 0.92) | <0.01* | 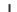 | 1.07 (1.01, 1.14) | 0.03*  | 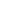 | 1.09 (1.02, 1.17) | <0.01* |
| Q5                | 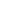 | 0.82 (0.78, 0.87) | <0.01* | 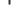 | 1.09 (1.01, 1.17) | 0.02*  | 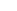 | 1.11 (1.03, 1.20) | <0.01* |
| <b>Arms</b>       |                                                                                   |                   |        |                                                                                   |                   |        |                                                                                     |                   |        |
| Q2                | 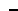 | 0.89 (0.84, 0.94) | <0.01* | 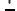 | 1.00 (0.93, 1.06) | 0.90   | 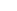 | 1.01 (0.95, 1.08) | 0.72   |
| Q3                | 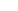 | 0.93 (0.87, 0.99) | 0.02*  | 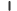 | 1.07 (1.00, 1.15) | 0.04*  | 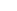 | 1.11 (1.03, 1.19) | <0.01* |
| Q4                | 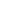 | 0.85 (0.80, 0.91) | <0.01* | 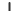 | 1.12 (1.03, 1.22) | <0.01* | 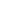 | 1.16 (1.06, 1.26) | <0.01* |
| Q5                | 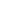 | 0.77 (0.72, 0.83) | <0.01* | 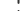 | 1.13 (1.02, 1.26) | 0.02*  | 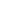 | 1.20 (1.08, 1.35) | <0.01* |
| <b>Legs</b>       |                                                                                   |                   |        |                                                                                   |                   |        |                                                                                     |                   |        |
| Q2                | 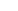 | 0.86 (0.81, 0.92) | <0.01* | 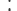 | 0.93 (0.87, 0.99) | 0.03*  | 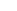 | 0.95 (0.88, 1.02) | 0.14   |
| Q3                | 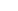 | 0.92 (0.85, 1.00) | 0.05   | 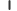 | 1.02 (0.93, 1.11) | 0.68   | 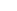 | 1.05 (0.95, 1.15) | 0.33   |
| Q4                | 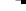 | 0.77 (0.70, 0.85) | <0.01* | 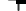 | 0.94 (0.84, 1.05) | 0.25   | 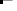 | 0.99 (0.89, 1.12) | 0.91   |
| Q5                | 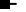 | 0.69 (0.62, 0.76) | <0.01* | 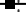 | 0.89 (0.78, 1.01) | 0.08   | 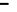 | 0.99 (0.86, 1.13) | 0.89   |
|                   | 0.7 1 1.3                                                                         |                   |        | 0.7 1 1.3                                                                         |                   |        | 0.7 1 1.3                                                                           |                   |        |

Supplement: Supplementary file 11 [file Data_Sheet_10.pdf]
