# Supplementary material for: Associations between total and regional fat-to-muscle mass ratio and fracture risk in elderly population: a prospective cohort study in UK Biobank
Source: Front Med (Lausanne). 2026 Jun 24;13:1830114. doi: 10.3389/fmed.2026.1830114 (PMC13341519; doi:10.3389/fmed.2026.1830114)
Supplement: Supplementary file 8 [file Data_Sheet_7.pdf]

FMR	Model 1	HR 1 (95% CI)	P1	Model 2	HR 2 (95% CI)	P2	Model 3	HR 3 (95% CI)	P3
**Whole body**									
Q2		0.87 (0.82, 0.94)	<0.01*		0.98 (0.92, 1.06)	0.68		0.99 (0.92, 1.07)	0.82
Q3		0.89 (0.83, 0.95)	<0.01*		1.05 (0.97, 1.13)	0.24		1.06 (0.98, 1.14)	0.15
Q4		0.79 (0.73, 0.85)	<0.01*		1.05 (0.96, 1.15)	0.28		1.09 (1.00, 1.19)	0.06
Q5		0.68 (0.63, 0.73)	<0.01*		1.02 (0.92, 1.14)	0.66		1.09 (0.98, 1.21)	0.10
**Trunk**									
Q2		0.86 (0.80, 0.91)	<0.01*		0.95 (0.89, 1.01)	0.11		0.96 (0.90, 1.02)	0.17
Q3		0.86 (0.81, 0.91)	<0.01*		1.02 (0.96, 1.09)	0.53		1.03 (0.97, 1.10)	0.34
Q4		0.82 (0.78, 0.88)	<0.01*		1.07 (1.00, 1.15)	0.06		1.09 (1.02, 1.17)	0.02*
Q5		0.77 (0.73, 0.82)	<0.01*		1.11 (1.02, 1.20)	0.01*		1.15 (1.06, 1.25)	<0.01*
**Arms**									
Q2		0.88 (0.82, 0.94)	<0.01*		1.02 (0.94, 1.09)	0.65		1.02 (0.95, 1.09)	0.64
Q3		0.89 (0.83, 0.96)	<0.01*		1.08 (1.00, 1.17)	0.06		1.09 (1.01, 1.17)	0.04*
Q4		0.80 (0.74, 0.86)	<0.01*		1.13 (1.03, 1.24)	<0.01*		1.16 (1.06, 1.27)	<0.01*
Q5		0.69 (0.64, 0.75)	<0.01*		1.13 (1.01, 1.27)	0.03*		1.19 (1.06, 1.34)	<0.01*
**Legs**									
Q2		0.85 (0.79, 0.92)	<0.01*		0.94 (0.87, 1.02)	0.12		0.95 (0.88, 1.03)	0.20
Q3		0.90 (0.82, 0.99)	0.04*		1.05 (0.94, 1.16)	0.40		1.07 (0.96, 1.19)	0.20
Q4		0.71 (0.64, 0.79)	<0.01*		0.94 (0.83, 1.06)	0.30		1.00 (0.88, 1.14)	0.97
Q5		0.61 (0.54, 0.68)	<0.01*		0.88 (0.76, 1.02)	0.09		0.98 (0.85, 1.14)	0.81
