# Supplementary material for: Associations between total and regional fat-to-muscle mass ratio and fracture risk in elderly population: a prospective cohort study in UK Biobank
Source: Front Med (Lausanne). 2026 Jun 24;13:1830114. doi: 10.3389/fmed.2026.1830114 (PMC13341519; doi:10.3389/fmed.2026.1830114)
Supplement: Supplementary file 9 [file Data_Sheet_8.pdf]

FMR	Model 1	HR 1 (95% CI)	P1	Model 2	HR 2 (95% CI)	P2	Model 3	HR 3 (95% CI)	P3
**Whole body**									
Q2		0.89 (0.84, 0.94)	<0.01*		0.97 (0.92, 1.03)	0.37		0.98 (0.93, 1.04)	0.46
Q3		0.90 (0.86, 0.96)	<0.01*		1.02 (0.96, 1.09)	0.44		1.03 (0.97, 1.10)	0.33
Q4		0.82 (0.78, 0.87)	<0.01*		1.03 (0.96, 1.10)	0.49		1.05 (0.98, 1.13)	0.15
Q5		0.76 (0.71, 0.81)	<0.01*		1.03 (0.94, 1.12)	0.50		1.08 (0.99, 1.18)	0.07
**Trunk**									
Q2		0.86 (0.82, 0.91)	<0.01*		0.93 (0.89, 0.98)	0.01*		0.94 (0.89, 0.99)	0.02*
Q3		0.87 (0.83, 0.92)	<0.01*		1.00 (0.95, 1.05)	0.99		1.01 (0.96, 1.06)	0.79
Q4		0.86 (0.82, 0.91)	<0.01*		1.05 (0.99, 1.11)	0.08		1.07 (1.01, 1.13)	0.03*
Q5		0.83 (0.79, 0.87)	<0.01*		1.09 (1.02, 1.16)	0.01*		1.12 (1.05, 1.20)	<0.01*
**Arms**									
Q2		0.90 (0.85, 0.95)	<0.01*		1.01 (0.95, 1.07)	0.76		1.01 (0.95, 1.07)	0.76
Q3		0.92 (0.87, 0.97)	<0.01*		1.06 (1.00, 1.13)	0.06		1.07 (1.00, 1.13)	0.04*
Q4		0.85 (0.80, 0.90)	<0.01*		1.12 (1.04, 1.21)	<0.01*		1.14 (1.06, 1.23)	<0.01*
Q5		0.77 (0.73, 0.82)	<0.01*		1.13 (1.03, 1.24)	0.01*		1.17 (1.07, 1.29)	<0.01*
**Legs**									
Q2		0.88 (0.83, 0.93)	<0.01*		0.94 (0.89, 1.00)	0.07		0.95 (0.90, 1.01)	0.13
Q3		0.92 (0.85, 0.99)	0.03*		1.01 (0.94, 1.10)	0.73		1.03 (0.95, 1.12)	0.41
Q4		0.76 (0.70, 0.83)	<0.01*		0.93 (0.84, 1.02)	0.13		0.98 (0.89, 1.08)	0.69
Q5		0.69 (0.63, 0.75)	<0.01*		0.89 (0.79, 1.00)	0.04*		0.97 (0.87, 1.09)	0.64
