# Supplementary material for: Associations between total and regional fat-to-muscle mass ratio and fracture risk in elderly population: a prospective cohort study in UK Biobank
Source: Front Med (Lausanne). 2026 Jun 24;13:1830114. doi: 10.3389/fmed.2026.1830114 (PMC13341519; doi:10.3389/fmed.2026.1830114)
Supplement: Supplementary file 12 [file Data_Sheet_11.pdf]

| FMR | Model a | SHR (a) (95% CI) | P (a) | Model b | HR (b) (95% CI) | P (b) | Model c | HR (c) (95% CI) | P (c) |
|---|---|---|---|---|---|---|---|---|---|
| **Whole body** | | | | | | | | | |
| Q2 | | 1.02 (1.00, 1.05) | 0.02* | | 0.97 (0.89, 1.07) | 0.57 | | 0.98 (0.89, 1.08) | 0.69 |
| Q3 | | 1.05 (1.03, 1.07) | <0.01* | | 1.02 (0.93, 1.12) | 0.67 | | 1.04 (0.94, 1.15) | 0.41 |
| Q4 | | 1.04 (1.01, 1.07) | 0.01* | | 1.05 (0.94, 1.18) | 0.40 | | 1.10 (0.97, 1.23) | 0.13 |
| Q5 | | 1.05 (1.02, 1.08) | <0.01* | | 1.05 (0.91, 1.20) | 0.53 | | 1.10 (0.95, 1.27) | 0.19 |
| **Trunk** | | | | | | | | | |
| Q2 | | 1.02 (0.99, 1.04) | 0.15 | | 0.92 (0.85, 1.00) | 0.04* | | 0.93 (0.85, 1.01) | 0.08 |
| Q3 | | 1.01 (0.99, 1.03) | 0.24 | | 1.01 (0.93, 1.10) | 0.83 | | 1.02 (0.94, 1.12) | 0.59 |
| Q4 | | 1.07 (1.04, 1.09) | <0.01* | | 1.10 (1.01, 1.20) | 0.04* | | 1.13 (1.03, 1.24) | 0.01* |
| Q5 | | 1.10 (1.08, 1.13) | <0.01* | | 1.10 (1.01, 1.22) | 0.04* | | 1.13 (1.02, 1.27) | 0.02* |
| **Arms** | | | | | | | | | |
| Q2 | | 1.05 (1.03, 1.07) | <0.01* | | 0.99 (0.91, 1.09) | 0.87 | | 1.00 (0.91, 1.10) | 0.98 |
| Q3 | | 1.12 (1.09, 1.14) | <0.01* | | 1.07 (0.97, 1.18) | 0.19 | | 1.10 (1.00, 1.22) | 0.06 |
| Q4 | | 1.10 (1.07, 1.13) | <0.01* | | 1.11 (0.98, 1.25) | 0.10 | | 1.18 (1.04, 1.33) | 0.01* |
| Q5 | | 1.12 (1.09, 1.15) | <0.01* | | 1.21 (1.04, 1.41) | 0.01* | | 1.32 (1.13, 1.54) | <0.01* |
| **Legs** | | | | | | | | | |
| Q2 | | 0.97 (0.95, 0.99) | <0.01* | | 0.94 (0.85, 1.04) | 0.22 | | 0.93 (0.84, 1.03) | 0.19 |
| Q3 | | 1.01 (0.98, 1.04) | 0.62 | | 1.03 (0.90, 1.17) | 0.71 | | 1.03 (0.89, 1.18) | 0.71 |
| Q4 | | 0.90 (0.87, 0.94) | <0.01* | | 0.97 (0.82, 1.14) | 0.68 | | 0.99 (0.84, 1.17) | 0.92 |
| Q5 | | 0.87 (0.84, 0.90) | <0.01* | | 0.96 (0.80, 1.16) | 0.68 | | 0.99 (0.82, 1.21) | 0.94 |
| | 0.7 1 1.3 | | | 0.7 1 1.3 | | | 0.7 1 1.3 | | |
